# Exploring Bio-Impedance Sensing for Intelligent Wearable Devices

**DOI:** 10.3390/bioengineering12050521

**Published:** 2025-05-14

**Authors:** Nafise Arabsalmani, Arman Ghouchani, Shahin Jafarabadi Ashtiani, Milad Zamani

**Affiliations:** 1 School of Electrical and Computer Engineering, College of Engineering, University of Tehran, Tehran 14395-515, Iran; n.a.salmani@ut.ac.ir (N.A.); sashtiani@ut.ac.ir (S.J.A.); 2 Department of Electrical and Computer Engineering, Aarhus University, 8000 Aarhus, Denmark; aghouchani@ece.au.dk

**Keywords:** bio-impedance, wearable devices, physiological changes, neuromorphic computing

## Abstract

The rapid growth of wearable technology has opened new possibilities for smart health-monitoring systems. Among various sensing methods, bio-impedance sensing has stood out as a powerful, non-invasive, and energy-efficient way to track physiological changes and gather important health information. This review looks at the basic principles behind bio-impedance sensing, how it is being built into wearable devices, and its use in healthcare and everyday wellness tracking. We examine recent progress in sensor design, signal processing, and machine learning, and show how these developments are making real-time health monitoring more effective. While bio-impedance systems offer many advantages, they also face challenges, particularly when it comes to making devices smaller, reducing power use, and improving the accuracy of collected data. One key issue is that analyzing bio-impedance signals often relies on complex digital signal processing, which can be both computationally heavy and energy-hungry. To address this, researchers are exploring the use of neuromorphic processors—hardware inspired by the way the human brain works. These processors use spiking neural networks (SNNs) and event-driven designs to process signals more efficiently, allowing bio-impedance sensors to pick up subtle physiological changes while using far less power. This not only extends battery life but also brings us closer to practical, long-lasting health-monitoring solutions. In this paper, we aim to connect recent engineering advances with real-world applications, highlighting how bio-impedance sensing could shape the next generation of intelligent wearable devices.

## 1. Introduction

### General Bio-Impedance Applications

Healthcare monitoring has become increasingly important in recent years due to the widespread adoption of smart devices such as smart rings, watches, and bracelets. This trend has resulted in a growing demand for wearable and portable devices. Numerous studies highlight the significance of wearable devices in continuously tracking vital health metrics, including vital signs and hemodynamic indicators like heart rate, respiratory rate, and blood pressure. These measurements are crucial for diagnosing respiratory and cardiovascular diseases, monitoring chronic conditions effectively, and reducing the need for hospitalization [1,2,3,4,5]. In addition to aiding in disease prevention, these devices empower patients to conveniently monitor their health status at home or by their bedside [6,7,8,9].

Bio-impedance measurement is a versatile and impactful tool in healthcare, with extensive applications ranging from cellular characterization [10,11,12] to body composition analysis [3,6,13]. Over the past century, it has demonstrated significant medical potential. Bio-Impedance Spectroscopy (BIS) provides valuable insights into the electrical properties of tissues and their relationship to physiological and pathological conditions. It can also serve as a diagnostic and predictive biomarker for various conditions, including respiratory and cardiovascular disorders, and cancer detection [11,14,15,16].

In bio-impedance measurement, a small-amplitude, safe alternating current (AC) is injected into the biological tissue, and the resulting voltage across the tissue is measured [9]. The complex impedance of the tissue is then calculated using techniques such as magnitude–phase detection or real–imaginary extraction, commonly referred to as IQ demodulation or synchronous detection.

**Figure 1:**
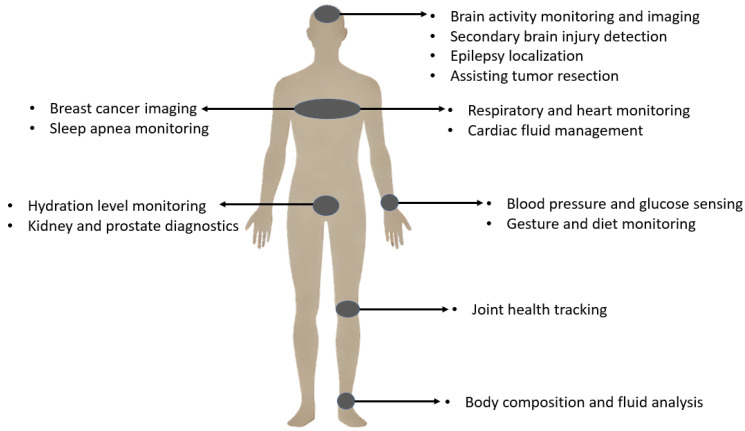
Bio-impedance applications.

Bio-impedance measurement has consequently found widespread applications across various medical fields and physiological monitoring scenarios. These diverse applications span multiple body systems and clinical conditions, mainly facilitated by non-invasive and wearable approaches. Figure 1 provides a visual overview highlighting key application areas of bio-impedance in the human body, while Table 1 offers a detailed categorization and comprehensive list of these uses, including relevant subcategories and references for further exploration.

**Figure 2:**
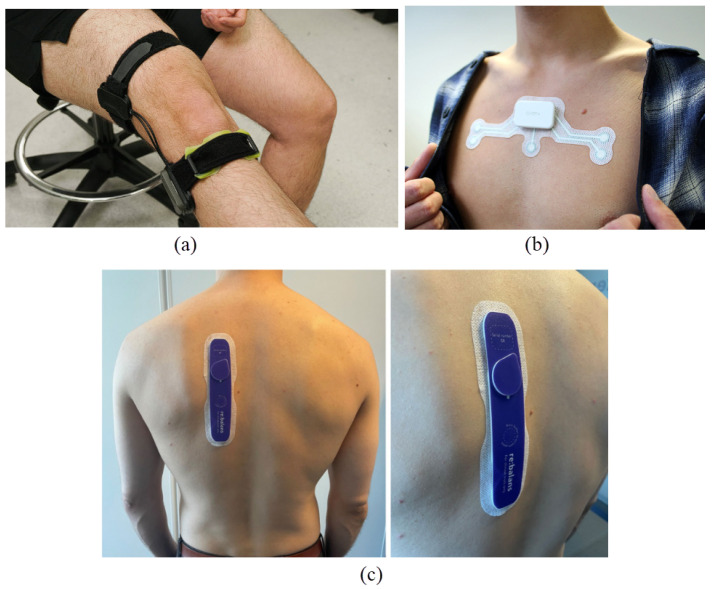
Wearable bio-impedance devices for (**a**) monitoring joint swelling and damage—source: Georgia Institute of Technology [17]—(**b**) detecting sleep apnea—photo: Onera Health Inc., Eindhoven, The Netherlands; source: IEEE Spectrum [18]—and (**c**) assessing fluid balance in end-stage renal disease patients [19].

Bio-impedance measurement can be performed either non-invasively or through integration into implantable devices. Bio-impedance measurement for non-invasive applications commonly utilizes wearable technologies that enable real-time monitoring of physiological biomarkers, facilitating the early detection of chronic conditions. Figure 2 shows examples of wearable bio-impedance devices and their placement on the body for different clinical applications. In recent years, wearable bio-impedance devices have attracted considerable attention due to their broad range of applications and ability to provide valuable insights into biological tissues’ physical and chemical properties.

While wearable bio-impedance is widely employed for monitoring body conditions, offering valuable insights as broadly demonstrated in Figure 1 and detailed in Table 1, its potential in non-invasive assessment and characterization of the brain is particularly significant for neuroscience research and clinical applications, aligning well with the focus on intelligent wearable devices. The electrical properties of brain tissue, such as resistance and reactance, are known to change in response to various physiological states and pathological conditions like ischemia, hemorrhage, edema, or cellular damage. This sensitivity makes wearable bio-impedance a promising tool for monitoring brain tissue status, detecting abnormalities, and potentially tracking the progression of neurological diseases using non-invasive means. Applying wearable bio-impedance for brain monitoring, however, presents specific challenges related to signal propagation through the skull, precise electrode placement on the scalp, and the interpretation of complex impedance changes within the brain tissue, which require dedicated review and analysis.

The remainder of this paper is organized as follows. Section 2 delves into the various applications of bio-impedance, specifically in the field of neuroscience and the study of brain diseases, including conditions such as epilepsy, stroke, ischemia, and secondary brain injuries. Section 3 provides the essential biological and electrical context by explaining tissues’ biological meaning and passive and electrical properties, focusing on brain tissue bio-impedance characteristics. Section 4 covers the critical technical aspects of measurement, discussing electrodes and electrical models for biological tissues. A detailed review of different bio-impedance measurement techniques, such as single-frequency and multi-frequency, is presented in Section 5. Section 6 then examines the hardware implementation aspects of bio-impedance measurement systems, detailing various approaches. Finally, Section 7 offers a comprehensive discussion on key considerations such as power consumption, electrode design, and digital implementation, concluding with future perspectives and emerging trends, particularly exploring the synergistic potential of neuromorphic computing for developing intelligent wearable bio-impedance measurement devices.

**Table 1:** Bio-impedance general applications.

Main Applications	Subcategories
Clinical Diagnostics and Therapeutic Monitoring	**Cardiovascular Health** - Heart Function Monitoring ∗ Heart Rate and Variability Monitoring [2,3,4,5,20] ∗ Stroke Volume and Cardiac Output Estimation [2,21] ∗ Blood Pressure Sensing [22] - Fluid Management in Cardiac Conditions ∗ Thoracic Fluid Accumulation Monitoring (Heart Failure) [2,3,4,21] ∗ Edema Detection [21] ∗ Assessing Fluid Balance During Dialysis [21,23] **Respiratory Health** - Lung Function and Ventilation Monitoring [1,5,6,8,21,24,25,26] - Pulmonary Edema Detection [21,27] - Respiratory and Sleep Apnea Monitoring [21,24,28] **Neurological Applications** - Brain Activity Monitoring and Imaging [15,29,30,31] - Epilepsy Localization and Monitoring [7,15,29,31] - Real-Time Detection of Secondary Brain Injuries [30] - Assisting Tumor Resection in Neurooncology [31] **Oncology** - Cancer Detection and Imaging ∗ Breast Cancer Detection and Imaging [14,32] ∗ Cancerous Tissue Differentiation [14,16,27,31] **Metabolic and Endocrine Disorders** - Glucose Estimation for Diabetes Management [33] **Body Composition and Fluid Analysis** - Body Composition Analysis (Fat, Muscle, Bone Estimation) [10,20,23,27,34] - Whole-Body Fluid and Hydration Monitoring ∗ Hydration Level Monitoring (e.g., Dialysis Patients) [21,23,25] ∗ Estimating Total Body Water (TBW) for Hydration Status [23,35] **Therapeutic Monitoring and Postoperative Care** - Tissue Recovery Monitoring [36] - Joint Health Monitoring [37] - Long-Term Monitoring for Chronic Conditions ∗ End-Stage Renal Disease [19,21,38] ∗ Ischemic Tissue Monitoring [11,30]
Advanced Bio-Impedance Techniques and Research Applications	**Electrical Impedance Tomography (EIT) and Imaging** - Lung Function and Ventilation Imaging [8,25,39] - Brain Imaging for Functional Mapping [15,29,30,31] - Breast Cancer Imaging [4,32] **Electrical Impedance Spectroscopy (EIS)** - Cancer Diagnosis and Tissue Characterization [14,39,40] - Tissue Differentiation and Modeling [14,16,27,41] - Advanced Impedance Modeling for Biological Tissues [42] **Cellular and Molecular Studies** - Cell Growth, Differentiation, and Viability Monitoring [40,42] - Neural Impedance and Potassium Ion Concentration Studies [15] - DNA Analysis **Biomaterials and Tissue Engineering** - Electrical Impedance Analysis of Scaffolds and Implants [40]

**Note:** Main applications categories are shown in bold.

## 2. Bio-Impedance Application in Neuroscience and Brain Diseases

### 2.1. Transcranial Impedance Changes (Epilepsy, Tumor, Ischemia, Blood Flow, Stroke)

Bio-impedance sensing has gained prominence in recent years due to its non-invasive, real-time, and cost-effective approach in neuroscience. Recent studies have demonstrated the applicability of BioZ in detecting, predicting, and monitoring various neurological conditions and brain diseases, such as stroke, epilepsy, and neurodegenerative disorders, intracranial bleeding, and cerebral edema [30,43].

Numerous neuroimaging techniques are available for brain tissue imaging, including X-ray, CT scans, MRI, and fMRI. Electrical Impedance Tomography (EIT) offers a promising alternative for neurological monitoring due to its real-time, non-invasive nature. This method reconstructs conductivity distributions and maps the impedance changes into the internal structure and activity of the brain. Approaches such as multi-frequency EIT (MFEIT) examine impedance differences between brain tissues using algorithms and machine learning models, enabling the distinction between ischemic and hemorrhagic strokes. In ischemic strokes, brain tissue impedance is higher due to reduced cerebral perfusion, whereas in hemorrhagic strokes, impedance is lower because of blood accumulation in the brain tissue [44,45].

Recent advancements in point-of-care (POC) technologies have highlighted the complementary roles of bio-impedance sensing, wearable Doppler ultrasound, functional near-infrared spectroscopy (fNIRS), and diffuse speckle pulsatile flowmetry (DSPF) in non-invasive physiological monitoring. Bio-impedance sensing offers a cost-effective and portable means to assess tissue and fluid characteristics, proving beneficial in applications such as body composition analysis and the monitoring of pulmonary congestion in heart failure patients [46]. Wearable Doppler ultrasound provides real-time hemodynamic assessment by measuring blood flow velocity, which is crucial for cardiovascular monitoring; however, its accuracy can be influenced by operator skill and anatomical variations [47]. fNIRS enables monitoring of cerebral oxygenation and hemodynamics, making it valuable for assessing brain function, though it is limited by shallow penetration depth and sensitivity to motion artifacts [48]. DSPF, an emerging optical technique, offers high temporal resolution in monitoring microvascular blood flow, which is particularly useful in evaluating peripheral artery disease, albeit with limitations in tissue penetration and susceptibility to optical property variations [49]. Integrating these modalities can enhance the accuracy and reliability of physiological assessments in various clinical settings.

Hannan et al. [50] demonstrate that epicortical and intracranial EIT (Electrical Impedance Tomography) can potentially track fast neural activity with millisecond resolution, and may reflect slow changes in brain tissue impedance during seizures. Their findings suggest that impedance tends to decrease during the spike phase of epileptiform discharges. This approach shows promise for detecting and localizing epileptic foci, particularly in cases of drug-resistant epilepsy. Early studies indicate that EIT could offer a safer and more accurate method for identifying epileptic foci compared to intracranial EEG, although further research is needed to fully establish its clinical utility [45].

Bio-impedance can also be an intraoperative real-time tumor delineation tool because tumorous tissues demonstrate lower impedance values than healthy white and gray matter. Abboud et al. [31] state that distinguishing gliomas from healthy surrounding tissues in neurooncology is essential, so intraoperative BioZ measurement enhances surgical precision and minimizes damage to healthy brain tissues.

Mivalt et al. [51] explore long-term impedance fluctuations in limbic structures and their relationship with brain extracellular space (ECS) volume changes. Therefore, bio-impedance measurement could be a potential biomarker for monitoring ECS dynamics and identifying sleep disorders and neurodegeneration. Meghdadi et al. [52] also investigate rheoencephalography (REG) as a non-invasive method to measure changes in intracranial impedance during sleep. Sleep quality and its stages are closely linked to cerebral blood flow (CBF), cerebrospinal fluid (CSF) movement, and overall intracranial fluid dynamics. CBF naturally declines during sleep, a change associated with the glymphatic system, which plays a key role in clearing waste metabolites from the brain. This process is thought to be important in many neurological diseases, including Alzheimer’s disease, where changes in CBF and CSF dynamics can occur before cognitive symptoms appear. Monitoring intracranial impedance fluctuations during sleep could therefore offer a way to identify early signs of brain disorders and guide prevention or treatment strategies. Given that bio-impedance sensing is low-cost and portable, it has strong potential for integration into wearable sleep-monitoring devices aimed at assessing brain health, particularly in aging populations.

Despite its advantages, bio-impedance sensing has several challenges, like signal attenuation due to the skull, low spatial resolution, and variability in electrode placements. To overcome these challenges, research is ongoing to use multimodal integration, for example, combining EIT with EEG, machine learning models, or AI reconstruction algorithms to enhance bio-impedance’s diagnostic precision [53].

### 2.2. Secondary Brain Injury (Ischemic (High-Impedance), Hemorrhagic (Low-Impedance), and Post-Surgical Monitoring)

Primary brain injuries are the result of an external traumatic brain injury (TBI), stroke, neurosurgical intervention, or ischemic events. In most cases, after hours to days, secondary brain injuries (SBIs) due to cerebral edema, ischemia, hypoxia, hemorrhage, inflammation, and hematoma will appear, and it is essential to identify primary etiology and manage it through medicine or surgical intervention. Every moment of delay in treatment can cause severe brain damage and lead to chronic morbidity and even mortality [30].

The impedance of brain tissue varies significantly depending on pathological changes. Wu et al. [43] report that the extracellular water content increases in cerebral edema, leading to a gradual decrease in impedance.

Ischemia occurs when a cerebral blood vessel is blocked due to injury or a clot, restricting the movement of extracellular fluid. Consequently, oxygen and glucose fail to reach the brain cells, resulting in ionic imbalance and neuronal swelling. Under these conditions, tissue impedance increases. According to Nalepa et al. [54], the impedance of ischemic tissue increased by 75% at frequencies below 10 Hz, and in the range from 1 kHz to 1 MHz, the impedance increased by 15%.

A hemorrhagic SBI resulting from cerebral bleeding exhibits significantly lower impedance than the surrounding tissues (0.7 S/m vs. 0.2 S/m, respectively) since blood has higher conductivity than brain tissue [30,55]. The impedance differences between ischemic, hemorrhagic, and healthy brain tissues make bio-impedance an effective tool for detection and monitoring SBIs. The ability to distinguish high-impedance ischemic events from low-impedance hemorrhagic events allows for prompt treatment without the need for repeated CT or MRI scans [30,43,53,56,57].

MRI and CT scans are the primary tools for monitoring patients with secondary brain injuries, and they provide static snapshots of brain status. Bio-impedance provides clinicians with real-time, continuous data on cerebral tissue changes, helping detect SBIs at early stages [30,45,56]. Also, intracranial bio-impedance is sensitive to blood volume changes in brain tissue, making it a potential biomarker for distinguishing between hemorrhage, ischemia, stroke, edema, and other brain injuries [30,43].

Increased intracranial pressure is a potential consequence of brain injuries [30,43,56,58]. Hawthorne et al. [59] investigate the potential of transcranial bio-impedance (TCB) as a non-invasive method for estimating intracranial pressure (ICP) in patients with secondary brain injuries. Traditional ICP monitoring has limitations in differentiating between various types of injuries, such as ischemic and hemorrhagic. Everitt et al. [30] present a novel intracranial bio-impedance monitoring (BIM) system that can detect changes in intracranial volume and distinguish between high-impedance (ischemic) and low-impedance (hemorrhagic) injuries, as well as differentiate focal (e.g., hemorrhage) from global (e.g., cerebral edema) brain events. This method significantly enhances patient outcomes in conditions like traumatic brain injury, stroke, hydrocephalus, and post-surgical monitoring.

Patients undergoing neurosurgical procedures for brain tumors, such as gliomas, may be at risk for complications including cerebral edema or tissue hemorrhage. These conditions can potentially contribute to secondary brain injury and significantly impact patient prognosis [43,60]. Changes in bio-impedance are associated with intracranial bleeding and intracranial pressure (ICP), making bio-impedance monitoring useful for real-time postoperative assessment and enabling timely interventions.

## 3. Bio-Impedance Biological Meaning and Properties

### 3.1. Passive Properties of Biological Tissues

In the early 1900s, researchers first demonstrated that the viability of cells could be assessed by measuring their electrical properties [61]. These electrical characteristics of biological tissues are influenced by various factors, including their physiological, morphological, and pathological states, as well as the frequency of the applied electrical signal [62,63]. Depending on the origin of electrical activity, these properties are broadly categorized as active (endogenous) or passive (exogenous). Active properties, also referred to as bioelectricity, result from ionic activity within cells, particularly nerve and muscle cells, and are typified by signals such as the electrocardiogram (ECG) from the heart and the electroencephalogram (EEG) from the brain. In contrast, passive properties arise as a response to external electrical stimulation and are the focus of this section [61,64].

Passive electrical properties of biological tissues are primarily characterized by bio-impedance, which describes the tissue’s ability to resist the flow of electrical current. This impedance can be evaluated by applying an external electrical signal, typically in the form of current or voltage, and measuring the tissue’s response. bio-impedance measurement systems employ electrodes to capture the excitation signal and the tissue response, where electrodes act as interfaces to facilitate the conversion between ionic charges in the tissue and electronic signals in the measurement system [65].

At the microscopic level, biological tissues are composed of cells with membranes, which are immersed in extracellular fluid (ECF) and enclose intracellular fluid (ICF) [66]. The cell membranes, which separate the extracellular and intracellular spaces, create two electrically conductive regions: the extracellular and intracellular compartments. Both ECF and ICF serve as resistive pathways for electrical current due to their ionic content. However, the cell membrane itself, composed of a lipid bilayer approximately 7 nm thick, exhibits insulating properties and behaves as a capacitor. This is attributed to its semi-permeable nature, which imparts a high capacitance and capacitive reactance, particularly at low and medium frequencies [61,67,68].

The conductive nature of ECF and ICF is central to bio-impedance measurements, as the flow of electrical current depends on the ionic concentration in these fluids. ECF is relatively uniform in composition, and its ionic makeup, shown in Table 2, primarily consists of sodium ions ($Na^{+}$) as the dominant cation [69]. In contrast, the ionic composition of ICF is more variable, depending on the specific cell type. For instance, in muscle cells, potassium ions ($K^{+}$) replace sodium ions as the most prevalent ion, due to the active transport of potassium into the cells via ion pumps. This variability in ICF composition complicates the direct measurement of its overall resistivity. Consequently, bio-impedance assessments provide insights into both the extracellular and intracellular environments, where the resistance and capacitance of tissues reflect the ionic content and membrane properties, respectively.

Because cell membranes exhibit capacitive behavior, extracellular fluid (ECF) resistance is ideally measured at very low frequencies. Practical impedance meters equipped with surface electrodes typically operate within a frequency range of 5–1000 kHz. To estimate the ECF resistance ($R_{e}$) at zero frequency and the total body water resistance ($R_{i}$) at infinite frequency, extrapolation techniques must be applied. Even if measurements at extremely low or high frequencies were possible, the relaxation mechanisms inherent in living tissues would prevent the impedance from reaching these theoretical resistances [23].

**Figure 3:**
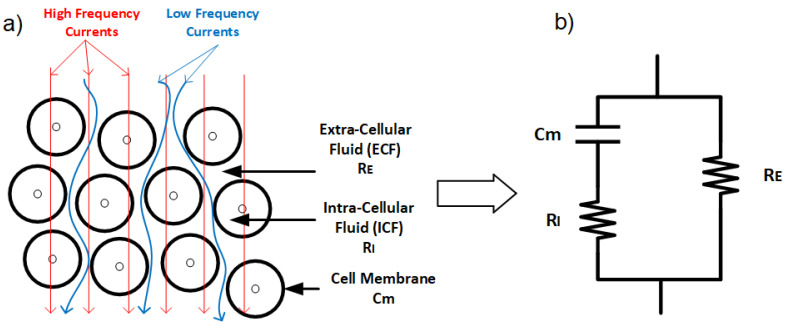
(**a**) Low-frequency current travels around the cell, while high-frequency current can penetrate cells. (**b**) Electrical model of the tissue with extracellular resistance ($R_{e}$), intracellular resistance ($R_{i}$), and conductance representing the cell membrane ($C_{m}$).

The electrical properties of cell membranes are primarily characterized by high capacitance and low, yet complex, conductivity. At direct current (DC) and low frequencies, electrical current predominantly flows around the cells, with minimal contribution from the intracellular regions. This is because the insulating properties of the cell membranes restrict current flow through the intracellular space. However, as frequency increases, the capacitive nature of the cell membranes allows alternating current (AC) to pass through them. Under these conditions, the influence of the membrane diminishes, and current flows more freely through the tissue, following the local ionic conductivity of both extracellular and intracellular regions (Figure 3) [65].

Although biological tissues can exhibit inductive properties, their contribution is negligible compared to resistance and reactance at frequencies below 10 MHz. For this reason, inductance is often disregarded in bio-impedance analyses [70]. The resulting complex electrical impedance of biological tissues, commonly referred to as bio-impedance, arises from the combined effects of tissue capacitance and conductance, both of which are frequency-dependent. This frequency dependence reflects the intricate structure and composition of biological tissues, as demonstrated by previous studies [62,71,72,73,74,75].

### 3.2. Electrical Properties of Biological Tissues

Bio-impedance is a technique used to evaluate the electrical properties of biological tissues, which vary across tissue types, such as bone and fat. In 1996, more than a century after initial investigations into the electrical properties of biological tissues in 1872 [64], Gabriel et al. [76] reported measurements of the dielectric properties of various biological tissues over a broad frequency range (10–20 GHz). These findings established the foundation for ongoing bio-impedance research across multiple applications.

Permittivity (ϵ) is one of most critical electrical property of biological tissues. In 1957, Schwan pioneered the study of the dielectric properties of biological tissues, observing that both permittivity (ϵ) and conductivity (σ) are frequency-dependent [77]. Permittivity represents the tissue’s ability to store electrical energy [65] and quantifies the tissue’s response to an applied electric field [71,78].

Under linear conditions in biological tissues, cell admittance (Y), cell impedance ($Z=\frac{1}{Y}$), and permittivity (ϵ) all convey equivalent information [65]. Permittivity is often presented as relative permittivity, which decreases as frequency increases due to the tissue’s limited ability to respond to rapid changes in the applied electric field [62,79,80].

In his analysis of biological tissue properties across a wide frequency range, Schwan identified three distinct dielectric dispersions: α-dispersion, β-dispersion, and γ-dispersion, which occur at low, radiofrequency, and microwave frequencies, respectively [63,77]:

1. α-dispersion (10 Hz to a few kHz): Generally associated with the diffusion processes of ionic species, related to tissue interfaces, such as membranes [77].
2. β-dispersion (1 kHz to several MHz): Attributable to the polarization of cellular membranes and large biological molecules, like proteins.
3. γ-dispersion (≥10 GHz): associated with the polarization of small molecules, like water molecules.

To study these properties, an alternating current at a single frequency or range of frequencies is applied to the tissue, and the tissue’s opposition to this current flow, or bio-impedance, is measured. Bio-impedance at any frequency is expressed as a complex number, with the real component representing resistance and the imaginary component representing reactance. Resistance is a measure of the tissue’s opposition to electrical current, while reactance indicates the ability of the tissue to store electrical current. Resistance is primarily associated with the fluids in the tissue, including dissolved ions, while capacitance is attributed to cell membranes. The membrane’s resistance is often negligible due to its small value (as illustrated in Figure 3b).

At low frequencies, the applied current primarily flows through the extracellular fluid (ECF) rather than penetrating the cells, enabling bio-impedance measurements to provide insights into the ECF. This occurs because cell membranes act as insulating barriers, establishing resistive pathways that prevent current from passing through the cells. In contrast, at high frequencies, the capacitive properties of the cell membranes become significant, allowing the current to penetrate the cells due to the high capacitance of the membrane, providing information on both intracellular and extracellular components [81] (Figure 3a). At very high frequencies, the current oscillates between cell membrane surfaces without sufficient time to establish resistive or capacitive pathways [73,82]. Resistance and reactance values from these measurements enable calculations of phase angle and magnitude, as discussed further in the next chapter [21].

### 3.3. Brain Tissue Bio-Impedance

Biological tissues exhibit significant variation in electrical properties, largely influenced by their fluid content. For instance, tissues like blood and brain are highly conductive, while lungs, skin, fat, and bone are poor conductors. Tissues such as liver, spleen, and muscle display intermediate conductivity. A comprehensive review of tissue-specific electrical properties across a broad frequency range is provided in the work of Gabriel et al. [71,76,83].

In the brain, impedance changes occur primarily through two mechanisms: slow impedance changes and fast neuronal depolarization. Slow changes, analogous to those observed in diffusion-weighted magnetic resonance imaging (MRI) or functional MRI (fMRI), unfold over tens of seconds. These are influenced by factors such as ischemia, where energy supply failure leads to water shifting from the extracellular to the intracellular space, increasing impedance significantly. Additionally, variations in blood flow, volume, and temperature during neural activity cause smaller decreases in impedance. Fast changes, on the other hand, are linked to rapid neuronal depolarization, where ion channel openings reduce bulk resistance, resulting in millisecond-scale impedance shifts [29].

**Figure 4:**
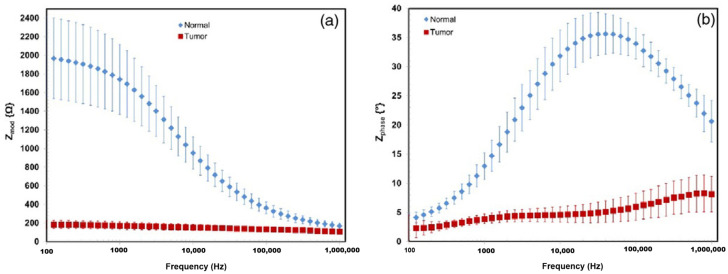
Impedance and phase presented as the averages of normal tissue and tumor tissue measurements from a representative case for the 100 Hz–1 MHz frequency range. (**a**) Shows the impedance modulus of the normal tissue in blue diamonds, with red squares indicating tumor tissue. (**b**) Shows the phase shift of normal and tumor tissue [85].

Tumors are abnormal tissue masses that proliferate at the expense of healthy surrounding tissue and lack a functional role. A distinguishing feature of tumors is their electrical properties, notably conductivity and permittivity, which often deviate significantly from those of normal tissue. For instance, Smith et al. [84] reported that liver tumors exhibited conductivity levels 6- to 7.5-fold higher and permittivity levels 2- to 5-fold higher compared to healthy liver tissue. However, these electrical characteristics are influenced by tumor type and developmental stage, as necrotic processes in the tumor core can affect conductivity. The dielectric contrast between tumor and normal tissues has facilitated the development of electrical impedance-based imaging systems for tumor detection and screening. At low frequencies, tumor tissues tend to demonstrate higher conductivity due to a reduced fraction of intact cells, whereas at high frequencies, the increased water content and irregular vascularization of tumor tissues result in enhanced conductivity, As illustrated in Figure 4, the impedance characteristics of normal and tumor tissues, measured ex vivo in human hepatic samples, demonstrate clear differences in frequency response [85]. These consistent electrical differences hold significant potential for clinical applications, particularly in non-invasive tumor detection [62].

Cerebral impedance changes have been extensively documented during epileptic seizures, as shown in various animal studies where seizures were induced using Metrazol, strychnine, and electrical stimulation [86,87]. Efron [86] highlighted impedance as a potential early indicator of seizure onset, observing a rapid rise in impedance preceding EEG abnormalities in 15% of cases. Van Harreveld [87] further suggested that the gradual increase in impedance during seizures might reflect processes that inherently limit seizure duration, drawing parallels with conductivity changes seen in cortical spreading depression. Consistent impedance variations were observed across brain regions, such as the hippocampus, amygdala, and cortex, in cat models during seizures. Notably, regions deeply involved in seizure activity displayed significant impedance shifts (10–12% above baseline), whereas regions with limited seizure propagation showed minimal changes [88]. These findings underscore the utility of impedance measurements in characterizing seizure dynamics and identifying regions of heightened seizure activity.

The skull’s inherently high resistance, which varies significantly in thickness among individuals, can attenuate impedance contrast by restricting current flow to the brain. Despite this limitation, substantial increases in brain impedance (ranging from 20% to 200%) have been documented in cerebral ischemic conditions [89]. This rise in impedance is primarily caused by the influx of ion-rich extracellular fluid into brain cells, leading to cellular swelling and tissue edema [90]. The resultant increase in impedance within the extracellular space has been well documented [29]. These impedance changes offer a promising method for the early detection of cerebral ischemia and stroke, particularly in cases of traumatic brain injury [53,91]. Continuous monitoring of impedance variations could thus play a pivotal role in improving diagnostic outcomes and enabling timely medical intervention.

**Figure 5:**
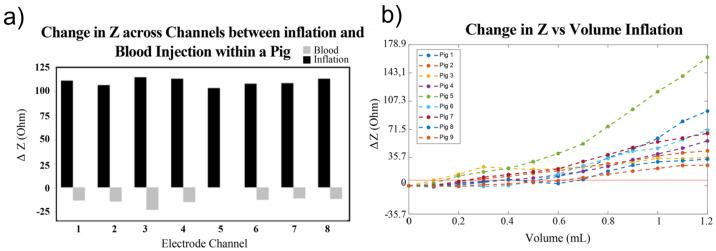
Comparison between inflation and blood injection on brain impedance. (**a**) ΔZ compared between injury types across all channels within a single pig. (**b**) Change in impedance from baseline of de-trended volume balloon inflation [30].

During ischemia, cells suffer from oxygen deprivation, leading to inflammation, a drop in pH, cell swelling, and eventual rupture, which alter the electrical properties of affected tissues [92]. In hemorrhagic events, active bleeding leads to blood pooling, with blood being more conductive (0.7 S/m) than surrounding brain tissue (0.2 S/m) [71]. Consequently, ischemic events reduce conductivity, raising impedance, while hemorrhagic events increase conductivity, lowering impedance [71,93]. In experiments on pigs [30], impedance changes during hemorrhagic and ischemic events have been investigated to assess intracranial volume (ICV) variations under controlled conditions involving blood injection and inflation, as shown in Figure 5a. The mean impedance shift from baseline demonstrated reliable detection of small volume changes (0.38 mL ± 0.19 mL) during inflation, as illustrated in Figure 5b, across nine pigs.

**Figure 6:**
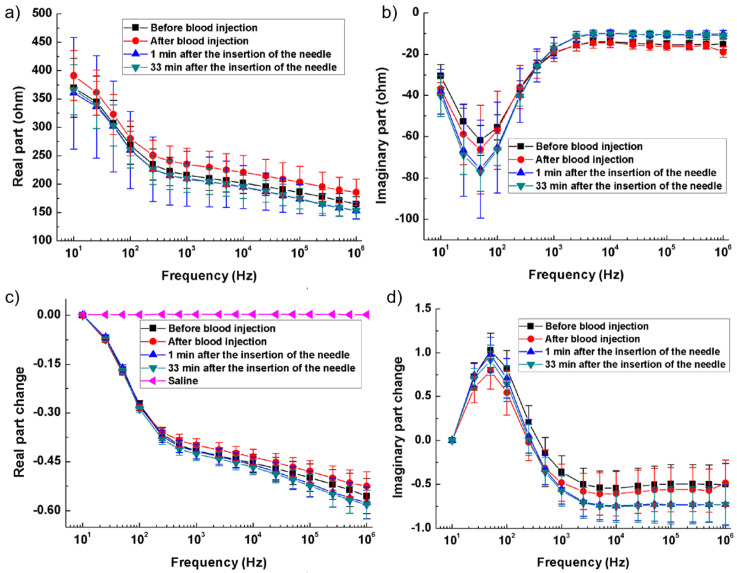
Brain impedance spectra of the ischemia group and its control. (**a**,**b**) Real and imaginary parts of the impedance spectra; (**c**,**d**) Changes in the real and imaginary parts of brain impedance relative to the impedance at 10 Hz [94].

In [94], with rabbit models simulating hemorrhagic and ischemic stroke, brain impedance was measured across frequencies from 10 Hz to 1 MHz, revealing significant differences between stroke-affected and healthy tissues. For hemorrhagic conditions, Figure 6a,b illustrate that the real part of impedance decreased from 10 Hz to 1 kHz, with the imaginary part peaking around 50 Hz. Relative changes in impedance (Figure 6c,d) show a 35% decrease in the real component from 10 Hz to 200 Hz and a further decline to −60% up to 1 MHz, while the imaginary component initially increased by 100%, then dropped by 150% from 100 Hz to 1 kHz before stabilizing. These findings highlight the distinct impedance characteristics of hemorrhagic and ischemic tissues, suggesting that specific frequency ranges (1 kHz–100 kHz and 500 kHz–1 MHz) could effectively differentiate stroke sub-types [94].

The following Table 3 presents a comparison of different phenomena and their effects on the quantity and variation of brain impedance. This includes variations in time, impedance changes, and specific brain regions affected, allowing for a detailed analysis of how each phenomenon uniquely impacts brain impedance characteristics.

## 4. Electrodes and Tissue Electrical Models

### 4.1. Bio-Impedance Electrical Models and Plots

Bio-impedance is measured using either the direct or indirect method. In the direct method, magnitude and phase are extracted by an impedance analyzer. These bench devices are suitable for laboratory setup measurements. Impedance analyzer ICs are also used in portable and wearable devices, but their applications are limited due to their bandwidth [42,96]. For example, AD5940 has high resolution and is applicable up to 200 KHz. MAX30001 is also suitable for low-frequency applications like respiration and cardiac monitoring.

Since abnormal tissues’ electrical properties differ from those of healthy ones, the direct method could be useful in neurooncology surgeries. It helps the surgeon intraoperatively distinguish epileptic zones during resection, which is crucial to locating and removing accurately, particularly in drug-resistance epileptic surgeries. Direct methods are also very beneficial for resectioning infiltrative tumors like gliomas, where determining the tumor margin is challenging for the surgeon. Therefore, direct methods in this field provide real-time feedback that complements pre-operative imaging techniques [31].

In the indirect method, an altered current is injected into the tissue, and the voltage drop across it is measured. The obtained bio-impedance data are analyzed using electrical and mathematical models. Accuracy in this method is limited to the selected electrical model [23,42,96]. Based on these models, common techniques such as IQ demodulation, FFT, and other signal-processing methods can be used to extract the magnitude and phase of the bio-impedance signal. The indirect method enables non-invasive measurements and real-time monitoring, making it highly suitable for wearable applications [40].

### 4.2. Electrical Models

From a historical perspective, in 1924, Frick investigated the electrical properties of the cell suspension [97] and introduced a mathematical framework for its analysis. One year later, in 1935, in collaboration with Morse, for the first time, they studied the resistive and capacitive behavior of blood cells in the frequency range of 800 Hz to 4.5 MHz [98] and presented an electrical model for it. In 1941, the Cole brothers presented the Cole–Cole model and plot to describe the electrical properties of biological tissues, the most widely used model. They introduced a generalization of Debye’s theory of dielectric relaxation. According to this theory, a single relaxation time is insufficient to describe the electrical behavior of complex biological tissues. To address this limitation, the Cole brothers proposed a distributed parameter for modeling, which replaces the classical assumption of a single relaxation time. The parameter α (alpha) ranges between 0 and 1. Based on this generalization, they developed a fractional-order model to characterize the impedance of biological tissues [99].

Shwan, in 1957, in studying the electrical properties of biological tissues, understood these properties like permittivity and conductivity change over frequency and introduced α,β,γ dispersion parameters, considering frequency ranges for characterizing dielectric properties of biological tissues.

Biological tissues have resistance-capacitance behavior. From a biological perspective, intracellular fluid (ICF) and extracellular fluid (ECF) have resistive behavior, and cell membranes have capacitive behavior. Bio-impedance measurement in multi-frequency, known as BIS, shows that currents pass through the ECF in low frequencies. In high frequencies, current penetrates the cell membrane and shows cell membrane capacitive and ICF/ECF resistive behavior. Therefore, we can consider an electric model for bio-impedance frequency response, as shown before in Figure 3 [20,21,38,40,41], that this electrical model can relate immittance spectra with physical and biological phenomena [42,100]. Hence, different and modified electrical models for biological tissues have been presented.

Biological tissues consist of various cell types arranged in complex structures and non-homogenous geometry. Therefore, tissue impedance is more complicated than simple combinations of resistor–capacitor modeling. Using fractional-order modeling based on fractional-order elements like constant phase elements (CPEs) is essential for modeling the non-linearity of tissue properties. The CPE models a phase shift that remains constant over frequencies. It accurately represents the biological tissues’ frequency behaviors arising from fractal and distributed nature [27,42,96].

**Figure 7:**
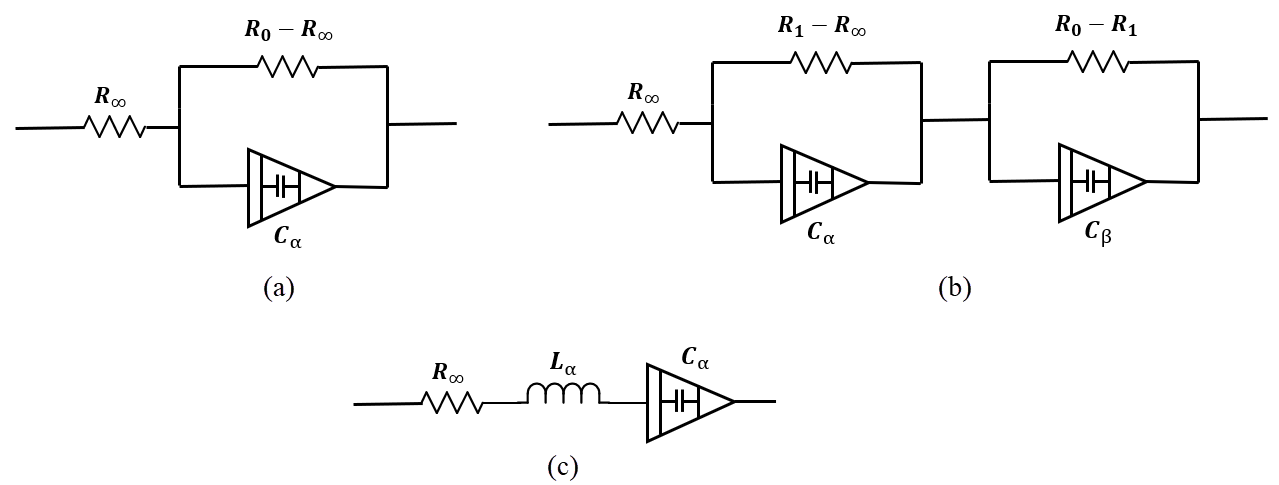
Bio-impedance circuit models. (**a**) Single-dispersion Cole model, (**b**) double-dispersion Cole model, (**c**) human respiratory system model.

Figure 7a shows a single dispersion Cole model containing a CPE element. In this model, one relaxation process dominates. Its impedance is

(1) $$Z=R_{\infty }+\frac{R_{0}-R_{\infty }}{1+s^{\alpha }C_{\alpha }\left(R_{0}-R_{\infty }\right)}$$

where:

- $R_{\infty }$: Resistance at high frequency,
- $R_{0}$: Resistance at low frequency,
- $C_{\alpha }$: Capacitance term associated with fractional-order behavior,
- α: Fractional exponent (0≤α≤1).

Figure 7b shows a double-dispersion Cole model, and Equation (2) shows its impedance.

(2) $$Z=R_{\infty }+\frac{R_{1}-R_{\infty }}{1+s^{\alpha_{1}}C_{\alpha_{1}}\left(R_{1}-R_{\infty }\right)}+\frac{R_{0}-R_{1}}{1+s^{\alpha_{2}}C_{\alpha_{2}}\left(R_{0}-R_{1}\right)}$$

where:

- $R_{\infty }$: Resistance at high frequency,
- $R_{1},R_{0}$: Resistances associated with the intermediate and low-frequency dispersions,
- $C_{\alpha_{1}},C_{\alpha_{2}}$: Capacitance terms for the two relaxation processes,
- $\alpha_{1},\alpha_{2}$: Fractional exponents for the two dispersions ($0\leq \alpha_{1},\alpha_{2}\leq 1$).

To date, many models have been presented. Some of them, like double-sell, Hayden, and wood moisture models, are specific to plant tissues, which are not the focus of this study. Also, in studies such as [101], depending on the desired accuracy, a dedicated model is presented to better match impedance spectrum data. In this study, the human respiratory system model has been presented to investigate the viscoelastic properties of the lungs. Figure 7c shows this model. Single- and double-dispersion Cole models are the most suitable options for brain tissue modeling, as they are widely used and accurately provide tissue electrical parameters. In brain injuries like ischemic reperfusion, we find a multi-factorial process in which modified Cole models can precisely model the brain tissue changes [102].

### 4.3. Data Visualization

Since tissue impedance varies over frequency, characterizing bio-impedance in a frequency range provides comprehensive information about its properties. Visually plotting the impedance variations provides a better insight into tissue properties. The Bode plot for bio-impedance will be obtained if the magnitude and phase are drawn in two distinct graphs vs. frequency. Considering Equation (3) for bio-impedance, the resistance and reactance parts are frequency-dependent because, as mentioned before, the current paths change through the tissue by frequency variations, which affect the resistance and reactance values. If the frequency parameter is omitted and reactance vs. resistance is plotted in an R-Xc plane, it gives a Nyquist plot, known as the Cole–Cole plot in bio-impedance studies, and widely used [42,100]. Impedance plotting tools like Bode and Nyquist are very useful for measured data analysis.

(3) Z=R+jX

**Figure 8:**
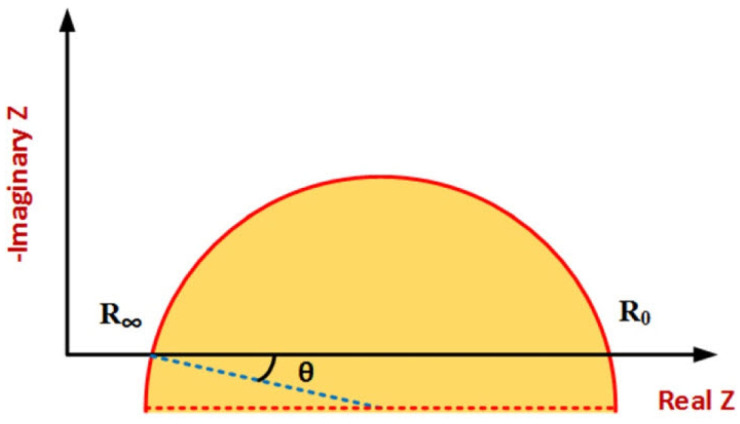
Typical Cole–Cole plot [96].

Figure 8 shows a typical Cole–Cole plot. Considering the classic Cole–Cole model, the locus of impedance spectroscopy data moves counterclockwise on a semi-circle curve by increasing frequency. Distinctive patterns and shapes in the plots provide useful information about tissue properties and frequency behavior [20,21,23].

The spectrum shows a single arc in the Cole–Cole plot in tissues where one-frequency dispersion is dominant. Tissues with multiple-frequency dispersion have several arcs in their plots. By considering electrode tissue impedance (ETI), both magnitude and phase are affected, resulting in more complex impedance spectrum data plots. The ETI effect on the impedance spectrum due to the electrodes’ design, configuration, type, and size is different and dominant in low frequencies. The most important application of impedance plotting is choosing suitable electrical modeling for data analysis using the indirect method [27,96,100].

### 4.4. Electrode Types

Different types of electrodes are used to monitor brain activities and functions. There are two main categories: scalp electrodes and intracranial electrodes. Electrode type, placement, and impedance variations significantly affect signal quality and measurement accuracy. Scalp electrodes are wearable and placed on the scalp non-invasively. For example, in brain imaging applications, they are often placed in a ring for data collection. Intracranial electrodes, which are implantable and placed directly on the surface of the brain tissue, are invasive and require surgery. They provide better spatial resolution compared to wearable (scalp) electrodes. These are used in cases where it is necessary to minimize the impact of the skull for increased accuracy and high resolution, or in specific procedures like oncology surgeries where mono-polar needle electrodes or intracerebral multi-contact electrodes may be utilized [29,30,31]. Wearable electrodes are further categorized into two types: wet and dry. Wet electrodes, commonly hydrogel-based, offer lower contact impedance and maintain higher signal quality. However, the hydrogel dries out over time and they are not convenient for daily use, making wet electrodes unsuitable for long-term monitoring. In contrast, dry electrodes are easy to use and reusable, making them suitable for long-term use. However, they typically provide less reliable signal quality due to higher impedance and are more sensitive to motion artifacts and variations in electrode placement. Table 4 summarizes the features and comparison of wearable electrodes [29,30,31].

### 4.5. Electrode Configuration

There are various types of electrode configurations. Electrode placement and configurations significantly impact the measurement results, making them a crucial factor in the design process [21]. Typically, the measurements are performed in two- or four-electrode configurations. They can be used in either invasive or non-invasive manners. Although some applications, such as tissue engineering [40], feature three-electrode configurations, this is not our primary focus.

**Figure 9:**
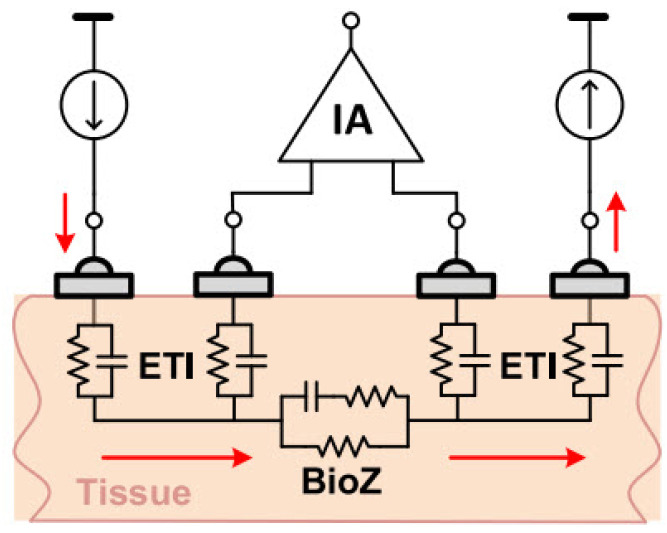
Four-electrode BioZ measurement configuration [20].

In a four-electrode or tetra-polar configuration, current is injected into the tissue through two electrodes. Then, the induced voltage in the tissue is measured by two other electrodes. As Figure 9 shows, distinct electrodes for voltage measurement result in negligible voltage drop through the electrode tissue impedance (ETI). Therefore, this configuration reduces the ETI error and is suitable for high-resolution measurements [20,34].

**Figure 10:**
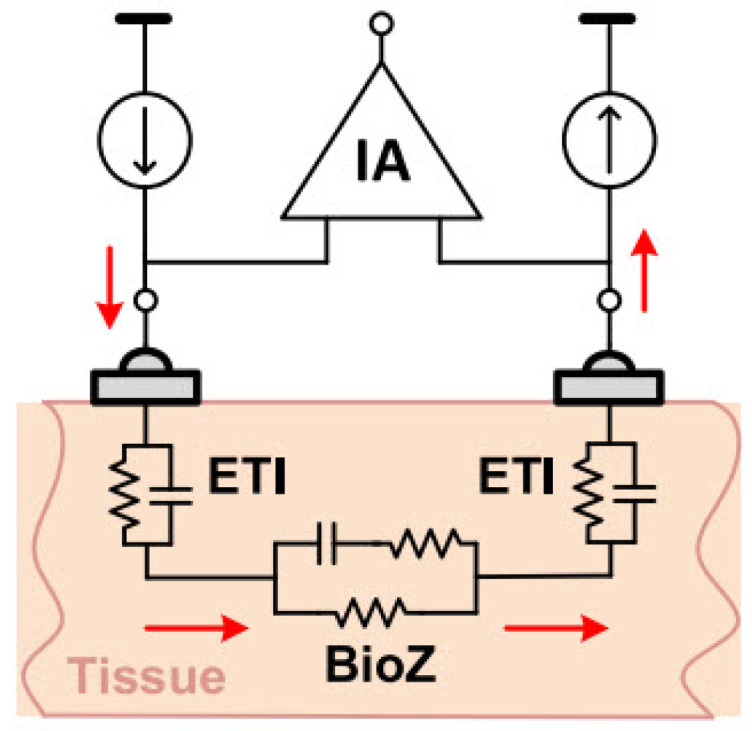
Two-electrode BioZ measurement configuration [20].

The two-electrode configuration is often preferred to simplify the design and reduce device size, making it well suited for wearable devices. Consequently, using dry electrodes in this configuration is ideal for daily use applications due to their simplicity, compactness, and user convenience. The impedance of dry electrodes is in the range of mega-ohms [20,34]. As illustrated in the Figure 10, measurement errors due to ETI can be significant, highlighting the need to investigate ETI and create an electrical model to understand its impact on BioZ measurement results.

The ETI value depends on many factors, including geometry, frequency, electrode type, and contact quality. Therefore, its impact varies across different applications and configurations [24].

### 4.6. Artifact Noises

The behavior of artifact noises caused by motion, contact resistance, or electrode placements depends on frequency. Consequently, multi-frequency measurement enables tracking and compensating artifacts, improving accuracy [27]. On the other hand, systems based on multi-frequency require more complex hardware and higher power consumption. Therefore, a trade-off must be considered in the design process. Also, we could detect and eliminate movement artifacts by integrating motion sensors with bio-impedance wearable devices [20,27].

## 5. Bio-Impedance Measurement Techniques

From a frequency perspective, bio-impedance systems are implemented in single-frequency or multi-frequency modes, depending on the application type and the desired specifications, each with advantages and disadvantages. Since the way electrical current flows through biological tissues depends on frequency, as discussed above, multi-frequency measurements are often used to obtain more detailed information about tissue properties [23]. Multi-frequency measurement can involve a limited number of frequencies or be performed as spectroscopy, in which case it is also known as Electrical Impedance Spectroscopy (EIS) or Bio-Impedance Spectroscopy (BIS) [21,23].

Single-frequency measurement is employed in applications where monitoring impedance changes with frequency is not intended, such as respiratory and cardiac output monitoring. This includes wearable devices that monitor vital signs and hemodynamic parameters, where low power consumption is a key design consideration [21,41]. Techniques like Electric Impedance Tomography (EIT) and Bio-Impedance Analysis (BIA) can also be implemented in either single- or multi-frequency modes, depending on the application.

### 5.1. Single-Frequency

Single-frequency measurement enables simple and efficient implementation. Designing a single-frequency signal generator is less complex compared to multi-frequency designs. Additionally, due to reduced harmonic demodulation, it offers higher accuracy and lower power consumption, making it more suitable for wearable devices [20]. In some applications, single-frequency measurement is also used to enhance stability and reduce the impact of noise [103].

The choice of frequency for design depends entirely on the application; however, single-frequency measurements typically use 50 kHz because phase angle measurement achieves higher accuracy at this frequency [20]. Cardiac and respiratory monitoring can be performed at a single frequency. For instance, this was conducted at 10 kHz in [104]. Single-frequency measurement is unsuitable for certain applications, such as monitoring brain activity or scenarios requiring tissue differentiation. In these cases, multi-frequency implementation is necessary [30,40].

### 5.2. Multi-Frequency

Electrical Impedance Spectroscopy (EIS) is an indirect method for characterizing biological systems by measuring their electrical properties and interpreting the measured impedance spectra using electrical models [42,96,100].

The multi-frequency method is recommended in applications where tissue characterization is required because it provides detailed tissue characteristics by exploring dispersion phenomena [96]. On the other hand, in some applications, such as studying the electrical properties of DNA, the use of EIS is essential. However, in this method, due to the record of bio-impedance at multiple and consecutive frequencies, a higher acquisition time is required than at a single frequency. It is also necessary to use more algorithms and steps for data analysis. This method provides a more comprehensive analysis of the tissue while requiring higher design complexity and power consumption [20,29,100]. The applications of multi-frequency measurements are wide-ranging, from single-cell analysis to whole-body diagnostics [100]. For example, brain tumor characterization using EIS has been performed in the frequency range of 10 Hz–5 MHz in [60].

### 5.3. Electric Impedance Tomography (EIT)

EIT is a medical imaging method. This method creates images using internal impedance measurements of organs via external electrodes. EIT can produce 2D or 3D images [6]. EIT is implemented in both single-frequency and multi-frequency modes. In the early systems used in 1984, implementations were single-frequency, usually at 50 kHz. Single-frequency systems were based on impedance changes over time. Due to their single-frequency nature, these systems are simpler to implement and are suitable for applications such as impedance changes in the lungs during breathing or fluid dynamics. However, single-frequency systems cannot analyze frequency-dependent impedance variations, making them ineffective for applications such as distinguishing tissue types or physiological details. Furthermore, they are useless in more complex analyses like brain imaging [29]. Since tissues have different spectral characteristics, they must be studied over a frequency spectrum. Therefore, EIT systems were implemented over time in multi-frequency (MFEIT) mode. For example, using EIT, lung function monitoring was performed in the range of 50 kHz–200 kHz [105]. These systems not only provide better characterization but also enable single-time imaging. Multi-frequency systems are more sensitive to noise compared to single-frequency systems. Also, image reconstruction requires more complex hardware and algorithms [21,29,40].

EIT systems are compact, making them portable and facilitating their use in clinical and non-clinical settings. Additionally, they can be utilized in wearable devices. However, since power consumption is a primary design consideration in wearable devices, they are typically designed and implemented as single-frequency systems [24,41], which cover limited applications.

The EIT technique is more cost-effective than other imaging methods, such as MRI and CT. It can also provide images of the tissue electrical characteristic changes within a few milliseconds, making it a suitable option for real-time studies, including monitoring rapid neuronal and physiological activities [29,30]. Another advantage of the EIT method is the capability for continuous monitoring, especially in chronic conditions. In contrast, other imaging methods like MRI provide a snapshot of the patient’s condition and require use in clinics by trained operators, while the EIT method is user-friendly [21]. Continuous monitoring of the patient’s condition can help prevent the deterioration of chronic conditions such as chronic heart failure and respiratory issues and reduce hospitalization. Additionally, continuous monitoring of brain function using EIT imaging can be highly beneficial for epilepsy or stroke patients [21].

EIT imaging has less spatial resolution than other imaging techniques like MRI, which limits its applications. Also, SNR is significantly important in this technique. For instance, in brain imaging applications, the skull has a much higher impedance than internal impedance variations. Furthermore, EIT imaging resolution is very sensitive to motion and noisy artifacts [21].

This technique utilizes a very small alternating electrical current, which makes it safe. It is also non-invasive and has no reported side effects, making it ideal for clinical and cognitive studies.

### 5.4. Bio-Impedance Spectroscopy (BIS/BIA)

BIS uses tissue electrical models to measure impedance across multiple frequencies, emphasizing zero and infinite resistances. In contrast, the SF-BIA (Single-frequency BIA) method is based on empirical equations and impedance measurements from wrist to ankle. It is typically used at a frequency of 50 kHz. Applications of these two methods include the non-invasive estimation of body fluid volumes, such as intracellular water (ICW), extracellular water (ECW), and total body water (TBW). The accuracy of these methods is limited by the electrical models and equations used. Accuracy can be improved with advanced algorithms or corrections based on body mass index (BMI) [23]. For instance, in [106], body composition measurement using BIA was performed at three frequencies: 6 kHz, 54 kHz, and 500 kHz. SF-BIA provides a good trade-off between accuracy and complexity [41].

Since SF-BIA operates at a single frequency, it cannot accurately separate intracellular and extracellular fluid compartments. At low frequencies, the current does not penetrate cell membranes, while at high frequencies, it passes through both intracellular and extracellular spaces without distinguishing between them. Therefore, SF-BIA suffers from inaccuracy in estimating intracellular fluids; however, it remains suitable for assessing total body water. This method is not sufficiently accurate in cases where the body fluid distribution is abnormal, such as in dialysis patients or individuals with edema. The BIS method is more complex than SF-BIA but provides good estimates of intracellular and extracellular fluids. Additionally, it remains suitable even in cases of abnormal fluid distribution [23]. Notably, BIA can also be implemented in a multi-frequency manner, which achieves good accuracy in estimating extracellular fluids and is less complex than BIS [41]. Depending on the application and system requirements, either method can be used.

## 6. Bio-Impedance Hardware Implementations and Mathematical Equations

For tissue impedance measurement, various methodologies can be employed, including both analog and digital domain techniques. Analog domain approaches include methods such as magnitude and phase demodulation or in-phase and quadrature (IQ) demodulation. Alternatively, digital domain techniques, such as direct digital processing, involve using only an instrumentation amplifier (IA) and an analog-to-digital converter (ADC) to directly convert the analog signal to a digital form. This enables computation of the real and imaginary components of the impedance directly within the digital domain.

### 6.1. Magnitude/Phase Measurement

The simplest approach to impedance measurement is the magnitude/phase technique, where the magnitude can be calculated using a peak detector [107] or through full-wave rectification followed by low-pass filtering of the measured sinusoidal voltage across the load [108]. Phase calculation requires a reference signal in-phase (0°) with the signal applied to the impedance under test. A phase detector compares square-wave versions of this reference signal with the measured signal across the impedance at each frequency, providing a measure of the impedance phase that can be further processed as described in [108]. This method requires two separate measurement channels—one for magnitude and one for phase—each with unique sources of potential error, and only the phase channel requires synchronization. For multi-frequency measurements, bandpass filters are necessary in this configuration, with a dedicated channel for each frequency. Impedance measurement systems generally employ AC voltage measurements following the injection of a constant-amplitude AC current (galvanostatic measurement) within medical safety limits. Alternatively, in biosensor applications, a voltage is applied, and the injected current is measured (potentiostatic measurement) [109,110,111]. However, voltage-output bio-interfacing is considered less safe due to the higher risk of tissue damage, as the current is uncontrolled when the load is variable [112].

**Figure 11:**
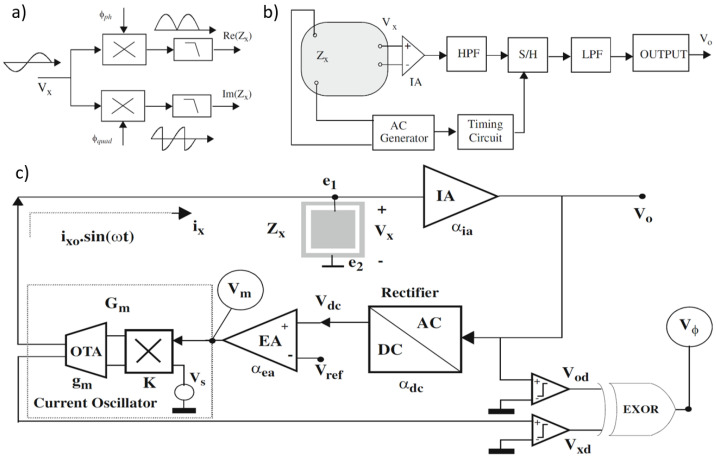
(**a**) Coherent demodulation. (**b**) Synchronous sampling. (**c**) Proposed circuit blocks for impedance sensing in [107]; magnitude and phase are obtained from signals Vm and V*ϕ*, respectively.

For measuring a given impedance Zx with known magnitude Zxo and phase ϕ, various methods are available, generally involving AC sources for excitation and either coherent demodulation [113] or synchronous sampling [114] for processing. Both approaches require synchronization between excitation and processing circuits to maximize noise performance, with high-pass (HP) and low-pass (LP) filters enhancing this further. Figure 11a,b illustrate block diagrams for these methods. A key drawback of the coherent demodulation [113] is the need for matching in-phase and quadrature channels to minimize phase errors. In contrast, synchronous sampling avoids dual channels and demodulation by selecting precise sampling times and incorporating an HP filter to reduce low-frequency noise and sampling interference.

In [107], circuits are proposed for measuring the impedance magnitude $Z_{xo}$ using an AC current excitation signal at a specified frequency. These circuits maintain a constant amplitude across the load ($V_{xo}=\mathrm{constant}$), satisfying the Potentiostat (Pstat) condition. The block diagram of the proposed circuit, shown in Figure 11c, includes key components such as an instrumentation amplifier (IA), a rectifier, an error amplifier, and a current oscillator with programmable output amplitude. The instrumentation amplifier provides a passband voltage gain $\alpha_{\mathrm{ia}}$. The rectifier functions as a full-wave peak detector, sensing the peak-to-peak voltage $V_{o}$ and producing a constant DC output $V_{\mathrm{dc}}$ with gain $\alpha_{\mathrm{dc}}$, such that $V_{\mathrm{dc}}=\alpha_{\mathrm{dc}}\alpha_{\mathrm{ia}}V_{xo}$. The error amplifier, with gain $\alpha_{\mathrm{ea}}$, compares this DC signal with a reference $V_{\mathrm{ref}}$ and amplifies any difference. The current oscillator generates the AC current to excite the load and consists of an external AC voltage source $V_{s}$, an Operational Transconductance Amplifier (OTA) with transconductance $g_{m}$, and a four-quadrant voltage multiplier with a constant *K*. The voltage generated by $V_{s}$, $V_{so}\sin\left(\omega t\right)$, is multiplied by $V_{m}$, and the resulting current is converted by the OTA. The equivalent transconductance from the magnitude voltage signal $V_{m}$ to the excitation current $i_{x}$ is given by $G_{m}=g_{m}\times V_{so}\times K.$ A simple analysis of the complete system yields an approximate expression for the voltage amplitude at $V_{x}$.

(4) $$V_{xo}=\frac{V_{\mathrm{ref}}}{\alpha_{\mathrm{ia}}\cdot \alpha_{\mathrm{dc}}}$$

when the following condition is satisfied:

(5) $$Z_{xo}G_{m}\alpha_{\mathrm{ea}}\alpha_{\mathrm{ia}}\alpha_{\mathrm{dc}}\gg 1$$

The condition is satisfied and defines the system’s closed-loop gain as $\alpha_{o}=Z_{xo}\times G_{m}\times \alpha_{\mathrm{ia}}\times \alpha_{\mathrm{dc}}\times \alpha_{\mathrm{ea}}.$ The voltage in Equation (4) remains constant if $\alpha_{\mathrm{ia}}$ and $\alpha_{\mathrm{dc}}$ also remain constant. Thus, the Potentiostat (Pstat) condition is met if the requirement in Equation (5) holds. Considering the relationship between the current $i_{x}$ and the magnitude voltage $V_{m}$ ($i_{xo}=G_{m}\cdot V_{m}$), the impedance magnitude $Z_{xo}$ can then be calculated.

(6) $$Z_{xo}=\frac{V_{xo}}{G_{m}}\cdot \frac{1}{V_{m}}$$

Equation (6) shows that from voltage Vm, the impedance magnitude Zxo can be calculated, since Vxo and Gm are known from Equation (4) and the design parameters.

The impedance phase can also be measured using the $V_{\phi }$ signal by assuming the input voltage oscillator $V_{s}$ is in phase with the current $i_{x}$. This signal can be squared or converted into a digital voltage signal $V_{\mathrm{xd}}$, serving as a time reference or sync signal. The $V_{o}$ voltage is also converted into a square waveform $V_{\mathrm{od}}$ using a voltage comparator. Feeding these two signals into an XOR gate generates a digital signal $V_{\phi }$, known as phase voltage, with a duty cycle *d* directly proportional to the phase being measured.

**Figure 12:**
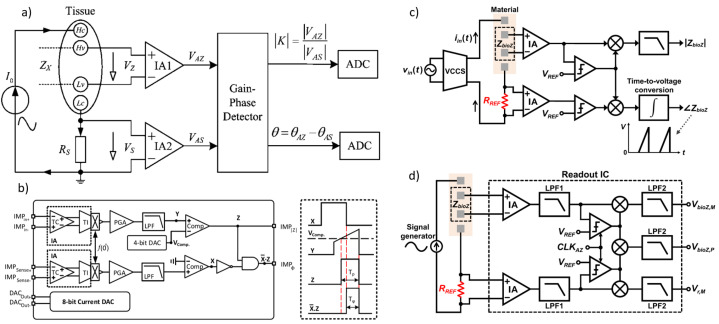
(**a**) Impedance measurement principle based on the magnitude-ratio and phase-difference detection (MRPDD) method [115]. (**b**) Architecture of the proposed bio-impedance measurement system [116] and example of internally generated signals. (**c**) Magnitude and phase measurement IC with a reference resistor [117]. (**d**) Magnitude and phase measurement IC with a reference resistor and an additional reference-magnitude measurement path [16].

In [115], the principle of the proposed BIS device based on the magnitude-ratio and phase-difference detection (MRPDD) method is described in Figure 12a. In the figure, $Z_{x}$ (hereafter, capital symbols represent complex quantities) represents the impedance of the tissue under measurement, and $R_{s}$ is a reference resistor connected in series with $Z_{x}$. A sinusoidal excitation current $I_{0}$ flows through $Z_{x}$ and $R_{s}$ via a pair of current electrodes $H_{c}$ and $L_{c}$. The voltage drop across $Z_{x}$ ($V_{z}$) is detected by a pair of voltage electrodes $H_{v}$ and $L_{v}$ and then amplified by instrumentation amplifier IA1, while the voltage drop across $R_{s}$ ($V_{s}$) is amplified by another instrumentation amplifier IA2.

Provided that the input impedances of IA1 and IA2 are infinite, there will be no current flowing through the two voltage electrodes, so the two output voltages of IA1 and IA2 can be expressed as follows:

(7) $$\left\{\begin{array}{c} V_{AZ}=A_{1}\cdot V_{Z}=A_{1}\cdot I_{0}\cdot Z_{x}\\ V_{AS}=A_{2}\cdot V_{S}=A_{2}\cdot I_{0}\cdot R_{s} \end{array}\right.$$

where $A_{1}$ and $A_{2}$ are the gains of IA1 and IA2, respectively. The gain-phase detector (GPD) compares $V_{AZ}$ and $V_{AS}$ and produces two DC outputs proportional to the magnitude ratio |K| and the phase difference θ of the input voltages:

(8) $$\left\{\begin{array}{c} \left|K\right|=\frac{|V_{AZ}|}{|V_{AS}|}\\ \theta =(\theta_{AZ}-\theta_{AS}) \end{array}\right.$$

From Equations (7) and (8), the unknown impedance $Z_{x}$ can be derived as follows:

(9) $$Z_{x}=R_{s}\cdot \frac{V_{Z}}{V_{S}}=R_{s}\cdot \frac{A_{2}}{A_{1}}\cdot \frac{V_{AZ}}{V_{AS}}=R_{s}\cdot \frac{A_{2}}{A_{1}}\cdot \left|K\right|e^{j\theta }.$$

If IA1 and IA2 are identically constructed with infinite input impedance, the gain ratio can be ideally set to $\frac{A_{2}}{A_{1}}=1$. Under this assumption, Equation (3) simplifies, yielding the measured impedance $Z_{m}$ as follows:

(10) $$Z_{m}=R_{s}\cdot \left|K\right|e^{j\theta }$$

where $R_{s}$ is a known standard resistor, and |K| and θ are the outputs of the GPD, sampled by ADCs. Equations (7)–(10) assume ideal conditions without considering disturbances. In practical applications, however, finite input impedance of IA1 and IA2, electrode contact impedance, and stray capacitance may introduce measurement errors.

In [116], Figure 12b illustrates the proposed bio-impedance readout channel, which includes two distinct paths: one for obtaining the magnitude of the bio-impedance and the other for extracting its phase. The implementation utilizes an 8-bit current DAC to inject a 2 kHz semi-ramp current into the heart tissue. This injected current complies with the IEC60601-1 medical standards [118], which specify the maximum amplitude and frequency for safe current injection. Adherence to this standard ensures safe bio-impedance measurements with minimal impact on heart tissue.

Figure 12b also shows the signals at various nodes in the amplitude and phase channels. The X waveform represents the PWM signal from the reference channel, originating from the voltage measured across the reference resistor and affected solely by the phase shift of the readout channel. The Y waveform is the input voltage to the comparator in the amplitude channel; it is a down-converted, amplitude-modulated signal of the bio-impedance semi-ramp that includes delays from the readout channel and the bio-impedance phase. The Z waveform is the amplitude channel output, with amplitude data encoded in the pulse width. The final waveform displays the difference between the reference signal and the amplitude channel’s PWM signal, allowing the phase data to be accurately reconstructed from its pulse width.

The phase readout channel is largely identical to the magnitude readout channel, with only minor differences. In the phase channel, the input signal is an ideal voltage generated by the injected current passing through a reference resistor, unaffected by the bio-impedance’s magnitude or phase. This input signal is then amplified, mixed, and filtered similarly to the magnitude readout channel. A comparator detects the zero-crossings by comparing the signal to zero.

The X signal represents the PWM output from the reference channel, related to the reference resistor and influenced only by phase shifts within the phase channel blocks. The Y signal is the input to the magnitude channel’s comparator and serves as the down-converted, magnitude-modulated signal of the bio-impedance semi-ramp. This signal includes delay effects from the readout channel and the bio-impedance phase. The Z signal, the magnitude channel output, encodes magnitude data within its pulse width. By comparing the PWM outputs of the phase and magnitude channels, a signal representing the phase of the bio-impedance can be derived.

In [117], as shown in Figure 12c, a clock derived from the reference resistor is used to detect the peak time of the measured bio-impedance (BioZ) signal, allowing magnitude values to be obtained at these peak times. This process incorporates a 90° phase shift of the reference clock. Unlike an I/Q demodulator, this configuration can capture delay information of the 90°-shifted clock using the signal generated from the reference resistor. This delay information is then used to compensate for errors from comparator delays and clock generator inaccuracies at each frequency, achieving a magnitude error of 1% and a phase error of less than 2°. However, the complexity of this circuit may make it susceptible to mismatches caused by parasitic elements. Additionally, current and capacitor-based integrators are needed to generate the 90°-shifted clock, which requires an extra comparator at high frequencies, leading to increased power consumption.

In [16], a simpler method for measuring magnitude uses a self-mixing full-wave rectifier, as shown in Figure 12c [108]. In this setup, the bio-impedance (BioZ) signal, amplified by an instrumentation amplifier (IA), is converted into a clock signal via a zero-crossing comparator. This clock then self-mixes with the BioZ signal, producing a rectified BioZ signal, which is subsequently low-pass-filtered to obtain the final magnitude measurement. The clock is also mixed with another clock generated from the reference resistor’s zero-crossing comparator. Both clocks pass through the IA, introducing similar delays and compensating for inherent IA delays and signal generator delays, leading to more accurate phase measurements. Despite this, errors due to IA nonlinearity and comparator delays may still introduce magnitude and phase errors of around 2% and 4°, respectively.

Figure 12d shows an improved structure to reduce these errors [119], which introduces an additional measurement path for the reference signal from the reference resistor. Since both paths share common components—IA, LPF1, chopper, and LPF2—their nonlinearity and comparator delay are matched, enabling mutual cancellation of these non-idealities in the magnitude and phase measurements. As a result, this approach achieves magnitude and phase errors of less than 1.1% and 2°, respectively. However, if the size difference between the reference resistor and BioZ is substantial, mismatches arise due to amplitude-dependent comparator delays and IA nonlinearity. To minimize these discrepancies, the reference resistor’s size can be adjusted to closely match the BioZ.

### 6.2. IQ Demodulator

Real and imaginary measurements are more complex than magnitude phase approach. A common approach is the sampling technique [120,121], where the known signal frequency, phase, and amplitude eliminate the need for Fourier transforms (FFTs). By sampling at precise instances when input frequency components peak and cross zero, real and imaginary components are obtained—this method is termed synchronous sampling (SS). Sampling in both half cycles of the period and averaging measurements removes offsets [121]. For multi-frequency signals, this approach allows real and imaginary component calculation at each frequency using a single channel [121], offering an advantage. However, achieving precise synchronization at high frequencies is challenging [120].

Synchronous detection (SD), also known as lock-in or phase-sensitive demodulation, is widely used for this measurement type and is well established [122,123,124]. The technique involves multiplying the sinusoidal voltage signal by in-phase (0°) and quadrature (90°) signals to calculate real and imaginary components at the target frequency. This locks onto a specific frequency component and demodulates it to DC, filtering out higher frequencies, including electrode DC voltages. A low-pass filter (LPF) rejects any components generated by multiplication, isolating the DC component proportional to the real or imaginary impedance value. This method imposes a frequency-selective property, eliminating the need for additional bandpass filters. While two channels are required, they are identical, which minimizes matching errors. For applications with slowly changing impedance, a single channel can be used, switching the demodulation signal between 0° and 90°. The filter’s cutoff frequency can be set by considering the lowest frequency of interest and the required settling time [125].

**Figure 13:**
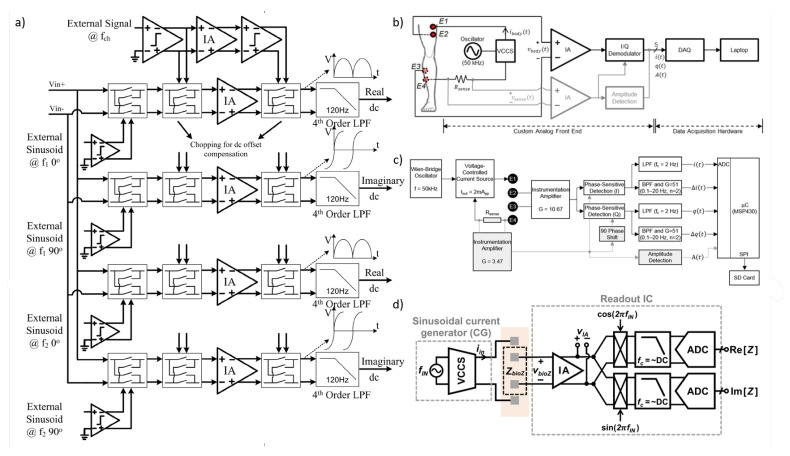
(**a**) System block diagram of the multi-frequency analog readout. The voltage versus time curves are examples of the outputs of the amplifiers when the offset is zero, the chopping is disabled, and input signal has no phase shift [39]. (**b**) Block diagram of the bio-impedance measurement system [128]. (**c**) Block diagram of the bio-impedance measurement system for local joint health assessment, where *i(t)* and *q(t)* represent the static (slowly varying on the order of hours to days), and *Δi(t)* and *Δq(t)* represent the dynamic (rapidly varying on the order of milli-seconds) bio-impedance components [129]. (**d**) Conventional I/Q system.

In [39], the block diagram of the analog readout is shown in Figure 13a. In a single frequency scenario, the SD modulator outputs for the real (V_SD_Re_) and imaginary (V_SD_IM_) channels are [124]

(11) $$V_{SD\_Re}=\frac{A}{2}\left[\cos\left(\phi_{1}\right)-\cos\left(2\omega_{1}t-\phi_{1}\right)\right]+C\sin\left(\omega_{1}t\right)$$

(12) $$V_{SD\_Im}=\frac{A}{2}\left[\cos\left(\phi_{1}+\frac{\pi }{2}\right)-\cos\left(2\omega_{1}t-\phi_{1}+\frac{\pi }{2}\right)\right]+C\sin\left(\omega_{1}t+\frac{\pi }{2}\right),$$

where *A* is the amplitude of the voltage across the electrodes, $\phi_{1}$ is the phase delay due to the impedance at angular frequency $\omega_{1}$, and *C* is the DC voltage of the electrode. For brevity, only the real part is considered in the following equations.

In a dual-frequency scenario with two frequency components, $f_{1}=\frac{\omega_{1}}{2\pi }$ (amplitude *A*) and $f_{2}=\frac{\omega_{2}}{2\pi }$ (amplitude *B*), the signal is demodulated at frequency $f_{1}$. The real part $V_{SD\_Re}$ becomes

(13) $$\begin{array}{cc} V_{SD\_Re}= & \frac{A}{2}\left[\cos\left(\phi_{1}\right)-\cos\left(2\omega_{1}t-\phi_{1}\right)\right]\\ \; & +\frac{B}{2}\left[\cos\left(\left(\omega_{1}-\omega_{2}\right)t+\phi_{2}\right)-\cos\left(\left(\omega_{1}+\omega_{2}\right)t-\phi_{2}\right)\right]+C\sin\left(\omega_{1}t\right), \end{array}$$

The component of interest is the DC value, given by $\frac{A}{2}\cos\left(\phi_{1}\right)$ for the real part and $\frac{A}{2}\cos\left(\phi_{1}+\frac{\pi }{2}\right)$ for the imaginary part.

Amplification and low-pass filtering can be combined as in [126]. However, to effectively reject common-mode signals at the electrodes, a high common-mode rejection ratio (CMRR) is necessary. This is achieved using a current-feedback IA [120,122,123] with digitally programmable gains of 10, 75, 140, and 200 *v*/*v*. The IA circuit topology for this design is detailed in [127]. To mitigate IA phase delay, demodulation is performed prior to amplification. A multistage clockless comparator [120] provides a square wave drive to the mixer switch (modulator), driven by a sinusoidal signal [39].

In [128], a custom analog front-end is used to acquire both static and dynamic bio-impedance measurements from the knee joint. In this study, "static" bio-impedance refers to the slowly varying impedance component, typically affected by structural changes in tissue volume (e.g., edema), while "dynamic" bio-impedance captures milliohm-level fluctuations in tissue impedance that are cardiosynchronous and correspond to the blood volume pulse. The measurement setup block diagram is shown in Figure 13b.

A 50 kHz, 2 mApp sinusoidal current is applied to the knee via electrodes E1 and E4, with the resulting voltage drop detected at electrodes E2 and E3. To account for any variability in the delivered current over time, the circuit also senses the injected current, denoted as $V_{\mathrm{sense}}\left(t\right)$. An I/Q demodulator, consisting of in-phase (i(t)) and quadrature (q(t)) phase-sensitive detection and filtering, is used to obtain both the in-phase and quadrature components of the measured voltage. Additionally, the signal $V_{\mathrm{sense}}\left(t\right)$ is passed through amplitude detection to obtain A(t), which is used to monitor the amplitude of the injected current.

In [129], as shown in Figure 13c, the objective of accurately quantifying edema and blood flow during the post-injury period necessitates a compact, energy-efficient system for bio-impedance measurements. To achieve this, a digitally assisted analog approach was employed, leveraging the strengths of both analog (low power consumption) and digital (programmability) domains. This system performs three main functions: (1) bio-impedance measurements, (2) calibration, and (3) preprocessing and feature extraction. Bio-impedance measurements from the body are conducted using a custom analog front-end built with discrete components.

All mentioned works [1,39,128,129] introduce the conventional structure. As depicted in Figure 13d, this structure is based on an I/Q demodulator, which is used to measure the real and imaginary components of the target impedance. The I/Q demodulation structure can be implemented with analog blocks, but it can also be implemented in the digital domain by oversampling the signal with an ADC and using digital multiplexers and filters [130]. The current signal generated by the sinusoidal current generator ($i_{\mathrm{in}}\left(t\right)$) can be represented as follows:

(14) $$i_{\mathrm{in}}\left(t\right)=I_{0}\cos\left(2\pi f_{\mathrm{IN}}t\right),$$

where $I_{0}$ and $f_{\mathrm{IN}}$ are the amplitude and frequency of the injected current signal, respectively. The current signal flows through the BioZ, and the voltage generated across the BioZ ($v_{\mathrm{bioZ}}\left(t\right)$), which is the input of the instrumentation amplifier (IA), can be represented as follows:

(15) $$v_{\mathrm{bioZ}}\left(t\right)=I_{0}\left|Z_{\mathrm{bio}}\right|\cos\left(2\pi f_{\mathrm{IN}}t+\theta_{\mathrm{bio}}\right),$$

where $|Z_{\mathrm{bio}}|$ and $\theta_{\mathrm{bio}}$ are the magnitude and phase of the BioZ, respectively. The received signal $v_{\mathrm{bioZ}}\left(t\right)$ is first amplified by the IA. Subsequently, when the amplified $v_{\mathrm{bioZ}}\left(t\right)$ signal ($v_{\mathrm{IA}}$) is multiplied by the in-phase and quadrature-phase signals, denoted as $\cos(2\pi f_{\mathrm{IN}}t)$ and $\sin(2\pi f_{\mathrm{IN}}t)$, the resulting voltages (vRe(t) and vIm(t)) are as follows:

(16) vRe(t)=A×I0|Zbio|×cos(2πfINt+θbio)×cos(2πfINt)=12AI0|Zbio|cos(θbio)+cos(2π2fINt+θbio)

(17) vIm(t)=A×I0|Zbio|×cos(2πfINt+θbio)×sin(2πfINt)=12AI0|Zbio|sin(θbio)+sin(2π2fINt+θbio)

where *A* is the IA gain. The high-frequency components, $\cos(2\pi 2f_{\mathrm{IN}}t+\theta_{\mathrm{bio}})$ and $\sin(2\pi 2f_{\mathrm{IN}}t+\theta_{\mathrm{bio}})$, located at $2f_{\mathrm{IN}}$ or higher, can be easily filtered out by subsequent low-pass filters (LPFs).

In practical circuit implementations, directly multiplying analog sinusoidal signals presents considerable challenges due to circuit complexity and high power consumption [131,132]. To overcome this, the chopping technique using a square wave clock is frequently applied. In this approach, the in-phase and quadrature-phase square signals are represented as a Fourier series of odd-harmonic tones. These tones are multiplied with $v_{\mathrm{IA}}\left(t\right)$ at frequency $f_{\mathrm{IN}}$ in the chopper, generating frequency components at $2Nf_{\mathrm{IN}}$, where *N* is a positive integer. If $i_{\mathrm{in}}\left(t\right)$ consists only of a pure sinusoidal waveform at frequency $f_{\mathrm{IN}}$, no harmonic folding occurs at the DC level. Thus, a low-pass filter (LPF) can effectively remove high-frequency terms introduced by the harmonic tones of the demodulation clocks. Consequently, the real component $|Z_{\mathrm{bio}}|\cos\left(\theta_{\mathrm{bio}}\right)$ and the imaginary component $|Z_{\mathrm{bio}}|\sin\left(\theta_{\mathrm{bio}}\right)$ are extracted, enabling calculation of the target impedance’s amplitude and phase.

### 6.3. Direct Digitization

**Figure 14:**
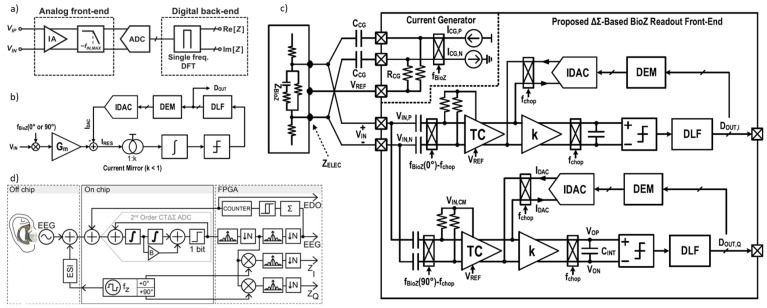
(**a**) Block diagrams of impedance-readout IC with direct sampling [16]. (**b**) Conceptual block diagram of the proposed BioZ readout. (**c**) Block diagram of the proposed BioZ readout [5]. (**d**) Ear EEG concept and AFE signal flow (channel block diagram) [138].

The direct sampling technique quantizes the impedance-modulated signal directly, bypassing the need for analog down-conversion to DC [133,134,135]. Figure 14a illustrates a block diagram of an impedance-readout IC employing direct sampling. This approach has several advantages over conventional analog demodulation, which involves down-conversion. One benefit is that it removes the need for a 90° phase-shifted analog signal, thereby simplifying system design. Furthermore, direct sampling enables the use of a wider-bandwidth low-pass filter (LPF), which can meet the ADC’s anti-aliasing requirements more easily.

In [16], LPF’s cutoff frequency can be set high enough to allow implementation with compact passive components, providing a fast settling time. By eliminating down-conversion and reducing LPF settling time, direct sampling enhances the system’s measurement throughput. However, there are trade-offs: direct sampling requires a high-speed ADC, which increases power consumption and restricts the measurement frequency range.

Another direct sampling technique, known as synchronous sampling, is described in [136]. In this method, a square-wave signal is injected into the target impedance, and the resulting impedance signal is amplified. The amplified signal is then sampled and quantized at the end of each period of the excitation current injection. This technique provides considerably higher throughput compared to conventional I/Q demodulation for impedance measurements, making it ideal for applications such as neural Electrical Impedance Tomography (EIT) [137], where rapid capture and analysis of neural activity are essential.

In [5], a direct digitization BioZ readout architecture is proposed to achieve both a wide dynamic range and low input-referred noise (Figure 14b). The wide dynamic range of this readout enables it to digitize BioZ signals without saturation, even when slow drifts in electrode impedance or motion artifacts are present. If these artifacts occur outside the bandwidth of interest (0.1–10 Hz), they can be filtered out; however, if they fall within this range, explicit artifact reduction techniques are necessary, particularly in ambulatory settings where motion artifacts can significantly impact tissue impedance. Although the proposed readout lacks explicit motion artifact reduction techniques, postprocessing software solutions can address this issue.

The architecture includes a first-order delta-sigma modulator [139] with a feedback IDAC and a digital loop filter (DLF) to closely track the input signal, minimizing residual current. This setup effectively suppresses input-dependent 1/f noise that increases with input signal amplitude. The BioZ readout also utilizes a single-ended IDAC [140] with no static current flow, reducing static power over a broad conversion range. A clocked averaging DEM is applied to the IDAC, suppressing both switching noise and 1/f noise without compromising circuit linearity. Additionally, since the IDAC shares the same reference current as the stimulation current source, reference noise is canceled without additional circuitry or power. As a result, the system’s noise performance is limited only by thermal noise.

Figure 14c presents the block diagram of the proposed system, which includes in-phase (I) and quadrature-phase (Q) BioZ RFEs and a current generator (CG). This CG produces a differential square wave current $I_{\mathrm{CG}}$ with adjustable amplitude (5 to 100 μAPK) and a tunable stimulation frequency $f_{\mathrm{BioZ}}$ ranging from 1 kHz to 1 MHz. On-chip pseudo-resistors (RCG) [141] and off-chip capacitors (CCG) are utilized for biasing and AC coupling, respectively. The input voltage $V_{\mathrm{IN}}$ in the two-electrode (2E) measurement setup can be expressed as follows:

(18) $$V_{\mathrm{IN}}=I_{\mathrm{CG}}\cdot \left(Z_{\mathrm{BioZ}}+2\cdot Z_{\mathrm{ELEC}}\right)$$

To remove the DC offset, $V_{\mathrm{IN}}$ is high-pass filtered. The input common-mode (CM) voltage $V_{\mathrm{IN},\mathrm{CM}}$ is controlled by negative feedback to ensure sufficient headroom for the IDAC, using a reference voltage $V_{\mathrm{REF}}$. Before entering the transconductance (TC) stage, $V_{\mathrm{IN}}$ is also premodulated at the chopping frequency $f_{\mathrm{hop}}$, reducing the TC stage’s required bandwidth and power [10]. A residual current is generated by subtracting the feedback current $I_{\mathrm{DAC}}$ from the TC stage output current, with the attenuated residual current accumulated by a Gm–C integrator to lower power consumption. The output $V_{0}$ can be expressed as follows:

(19) $$V_{0}=V_{\mathrm{OP}}-V_{\mathrm{ON}}=\frac{k}{C_{\mathrm{INT}}}\cdot \int \left(G_{m}\cdot V_{\mathrm{IN}}-I_{\mathrm{DAC}}\right)\;dt$$

where *k* is the current gain (less than 1), $G_{m}$ is the TC stage transconductance, and $C_{\mathrm{INT}}$ is the integrator capacitor’s capacitance. A phase shift between modulation and demodulation clocks may cause voltage spikes at the TC stage input, which cannot be compensated by the limited-loop bandwidth of the IDAC feedback. Choosing $f_{\mathrm{BioZ}}$ carefully can reduce this phase shift. The Gm–C filter’s low-pass characteristics help filter high-frequency spikes, protecting subsequent blocks. A clocked comparator provides a 1-bit output to control the IDAC. If this output is used to directly adjust the IDAC, coarse resolution could result in large residual currents; hence, an IIR-based digital loop filter (DLF) is used to refine residual current and increase loop gain [140], enabling precise IDAC control.

In [138], the wearable ear-EEG systems are depicted that use the front-end with share channel for both EEG and electrode-skin impedance (ESI) recordings, converting analog to digital domain directly using a 1024× oversampled 2nd order Continuous Time Delta Sigma (CTΔ*Σ*) ADC and process on a FPGA. Figure 14d shows the channel block diagram of this work.

## 7. Discussion and Perspective

As previously mentioned, bio-impedance has a wide range of applications. Due to its potential in various fields, this method’s non-invasive nature, real-time functionality, and ability to enable remote monitoring make it an excellent candidate for health monitoring. These sensors allow long-term use and are convenient for users using medical-grade intelligent wearables. With advancements in this area, these devices now provide precise data suitable for medical diagnosis, treatment, or continuous health management. Additionally, with the growing popularity of electronic gadgets and wearable devices such as smartphones, smartwatches, and bracelets, more individuals are adopting health-monitoring systems integrated into these technologies to support early disease detection and reduce treatment expenses [21]. Future bio-impedance devices aim to enhance resolution and improve efficiency. Next-generation wearables focus on compact designs, low power consumption, high accuracy, and longer battery life [21,38].

### 7.1. Power Consumption

Wearable devices must be designed for very-low-power operation, whether powered by a battery or through energy harvesting. This necessity arises from the demand for long-lasting performance and a compact form factor for integrating wearable gadgets [30]. The next generation of these devices is advancing towards multimodal sensors to improve accuracy and efficiency. Consequently, advanced IC technologies (65/55 nm CMOS) are essential for these designs, requiring low supply voltage and ultra-low power consumption.

As power consumption is the primary constraint in emerging wearable devices, reducing it enhances their practical usability [20,21,24,41]. Power management techniques can also further optimize energy usage [38]. Additionally, the choice of measurement technique directly affects power consumption. As noted earlier, FFT-based systems achieve reduced power consumption with minimal hardware requirements [35]. Given the importance of minimizing power consumption in future wearable devices, designs are increasingly expected to prioritize predominantly digital implementations.

### 7.2. Electrode Design

Another critical aspect of designing bio-impedance wearable devices is the development of electrodes. These devices are expected to monitor bio-impedance continuously and in real time. Designing gel-free (dry) electrodes and incorporating a two-electrode structure is essential for comfortable and long-term use. However, dry electrodes are significantly more susceptible to motion artifacts and noise than wet electrodes. Additionally, the electrodes’ material, size, and shape are crucial to their effectiveness. Therefore, the electrodes in wearable devices must be designed flexibly, depending on the intended application. Among these, bio-compatible materials and proper electrode placement are particularly important for ensuring reliable contact between the electrode and the skin. As a result, trends in electrode design for wearable applications are moving towards flexible and stretchable designs capable of adapting to the body’s movements. For instance, electrodes can be integrated into textiles [41], or nanowires can be used for fabrication [142]. Such designs can also be embedded in smart garments [21,38,142].

Precision in two-dry-electrode structures remains a significant challenge. In recent years, many designs have adopted four-electrode structures to achieve higher measurement accuracy and resolution [20,21,38]. Miniaturization exacerbates this issue because smaller electrodes result in higher impedance, thereby increasing errors caused by electrode tissue impedance (ETI) [27]. In two-electrode configurations, ETI and other environmental noises introduce measurement errors that necessitate elimination through advanced techniques.

Hybrid structures can be employed [34] to minimize device dimensions without sacrificing accuracy. However, even with these approaches, miniaturization remains limited [27]. Emerging systems also use digital active electrodes (AEs) instead of passive ones. In these designs, the instrumentation amplifier (IA) is integrated with the electrode, offering digital output and a digital backend. This configuration significantly reduces errors caused by environmental noise by eliminating wiring between the electrode and the IA. Additionally, it provides greater scalability, enabling more AEs to be added to the data bus. Nevertheless, digital AEs are more complex than passive ones and are sensitive to mismatches [20].

Electrode patterns also warrant attention. Various structures, such as inter-digital designs, can enhance selectivity and sensitivity. Depending on the application, materials such as gold, silver, or nanomaterials like carbon nanotube and graphene can be used for substrates or deposition layers. The delicate interplay between nanomaterials and bio-molecules can significantly improve biosensor performance. Electrodes can also be implemented in array configurations, each requiring specific design considerations [143].

### 7.3. Digital Implementation

The noise issue is particularly significant in both two-electrode structures and very-low-power designs, affecting signal integrity. Furthermore, the rate of bio-impedance changes is generally less than 10 Hz and is strongly influenced by low-frequency (flicker) noise. The baseline impedance value is also significantly higher than bio-impedance changes, requiring a dynamic range exceeding 100 dB. Achieving high impedance measurement accuracy is crucial for chronic conditions [21]. Therefore, techniques such as digital signal processing must be used to eliminate errors.

Multi-frequency measurements, such as Electrical Impedance Spectroscopy (EIS), are more susceptible to noise. As a result, advanced noise reduction methods, including digital and adaptive filtering, are essential for improving the system’s signal-to-noise ratio (SNR) [100]. As previously mentioned, achieving high accuracy and resolution in bio-impedance instruments requires more precise electrical modeling, such as fractional-order modeling. Optimization algorithms are necessary for parameter extraction from bio-impedance data, which rely on digital platforms for implementation [42].

Fast Fourier Transform (FFT)-based systems are suitable for applications that demand high-accuracy measurements. However, these systems involve significant signal-processing complexity, which is implemented digitally. FFT-based techniques offer the potential for fully digital implementations [35,144]. So, the design approach for bio-impedance wearable devices is increasingly shifting toward digital or fully digital frameworks [20,24].

The requirement for 24-h continuous patient monitoring generates a substantial volume of data. Analyzing these data necessitates appropriate computational and statistical tools. Machine learning algorithms, which have demonstrated their effectiveness in studies on sleep apnea diagnosis, dehydration in pediatrics, and hemodialysis [21], are among the suitable options. These methods are implemented and executed digitally. Additionally, analog or digital signal-processing calibrations are valuable for ensuring precise measurements. Employing machine learning techniques, including Artificial Neural Networks (ANNs) and deep learning, can further enhance accuracy [34,38,96,143,144]. ANNs are particularly effective in applications requiring classification and decision-making, such as detecting activity types [42,144].

Machine learning (ML) and deep learning (DL) models have significantly enhanced bio-impedance-based applications such as body composition analysis, cuffless blood pressure measurement, and Electrical Impedance Tomography (EIT). These models facilitate more accurate and real-time diagnostics through deep autoencoders and hybrid reconstruction networks. Moreover, AI can eliminate artifacts in bio-impedance signals and optimize electrode placement in medical imaging. Despite these advancements in integrating AI into bio-impedance applications, challenges persist, including data variability caused by electrode placements and analyzers. Additionally, a high volume of data is essential for effectively training AI models, which is particularly challenging to obtain in human-centric datasets. Proposed solutions to these challenges include open-source databases and synthetic data generation methods like Generative Adversarial Networks (GANs). Another hurdle is the interpretability of AI models, which has seen some improvement through explainable artificial intelligence (XAI), but further enhancement of model explanation and usability is still required [145].

Emerging intelligent wearable devices require continuous data processing, making power consumption, processing speed, and system adaptability crucial. Computations must be performed closer to the sensor to address these challenges, reducing power usage and latency.

Instead of transmitting large amounts of raw data to cloud-based servers, edge computing processes information directly on the device, optimizing efficiency by reducing latency, bandwidth usage, and energy consumption. However, conventional von Neumann architectures suffer from high power consumption and latency due to separating memory and processing units.

Neuromorphic computing offers an efficient solution with low power consumption, parallel processing, and adaptability. Based on spiking neural networks (SNNs), it mimics the brain’s event-driven processing, making it ideal for real-time sensory data analysis in extreme edge applications. Integrating neuromorphic computing into extreme edge processing revolutionizes intelligent wearable devices by offering an energy-efficient, brain-inspired approach to processing sensory data in real-time.

Advancements in memory technology and neuromorphic hardware such as memristive devices and chips like Intel’s Loihi and IBM’s TrueNorth drive continuous adaptive learning, real-time health monitoring, and personalized diagnostics with minimal power consumption. Thus, neuromorphic edge processing is shaping the future of intelligent wearable technologies, enabling more efficient and personalized always-on wearables [146].

### 7.4. Toward Intelligent Wearable Bio-Impedance Measurement Devices

Bio-impedance, which quantifies the opposition to electrical current flow through biological tissues, has emerged as a versatile tool for understanding and monitoring physiological processes. By integrating bio-impedance data with other bio-signals, researchers can obtain deeper insights into complex biological systems, enabling the extraction of more meaningful and comprehensive information compared to using single modalities alone.

One significant application of bio-impedance is in epilepsy monitoring and diagnosis. Electrical Impedance Tomography (EIT), a non-invasive bio-impedance imaging technique, has shown considerable promise in detecting impedance changes associated with seizure activity. When combined with intracranial electroencephalography (EEG), which records brain electrical activity, EIT enhances the localization of seizure foci by providing both spatial and temporal information. EIT can visualize the spatial propagation of seizure activity, while EEG offers precise temporal data on seizure onset and progression. This synergy allows for a more detailed understanding of seizure dynamics, which is invaluable for guiding surgical interventions and optimizing treatment strategies. For instance, studies utilizing EIT in conjunction with depth electrodes in rat models demonstrated the ability to image fast electrical changes during seizures, correlating impedance variations with seizure propagation patterns observed on stereoelectroencephalography (SEEG). Further advancements, such as multi-frequency EIT systems, have improved its utility by enabling simultaneous acquisition of data across multiple frequencies. This approach enhances sensitivity to subtle impedance changes during epileptic events and provides richer information about tissue properties.

Another area where bio-impedance measurements have proven valuable is the study of cerebral blood flow (CBF) and fluid dynamics during sleep. Rheoencephalography (REG), a non-invasive technique that monitors impedance changes across the head, primarily reflects variations in cerebral blood volume and flow. Combining REG with polysomnography (PSG), which includes EEG and other physiological signals, facilitates a comprehensive assessment of cerebral hemodynamics during different sleep stages. Studies have shown that REG amplitude, particularly peak-to-trough and envelope measurements, correlates strongly with CBF changes, as demonstrated during controlled breathing maneuvers that modulate cerebral blood flow. Sleep stage-specific variations in impedance have also been observed, with lower impedance values reported during non-REM sleep stages N1 and N2, indicative of decreased cerebral blood volume. Furthermore, sleep stage N3, characterized by slow-wave sleep, exhibits the lowest variability in REG parameters such as frequency and amplitude, suggesting a more stable hemodynamic state during this stage. Analyzing these impedance variations and their variability provides deeper insights into sleep physiology and the hemodynamic changes underlying different sleep stages, contributing to a better understanding of sleep-related disorders.

Impedance measurements also play a critical role in elucidating the dynamics of the brain’s extracellular space (ECS), a compartment essential for neuronal signaling and overall brain function. The ECS volume fraction, which significantly influences tissue impedance, can be monitored to track changes in ECS size across different brain states. Studies have demonstrated that impedance in limbic structures oscillates over various timescales, including ultradian and circadian rhythms, and these oscillations correlate with transitions between wakefulness, non-REM (NREM) sleep, and REM sleep. Such findings suggest a strong relationship between ECS dynamics and sleep–wake regulation. Additionally, impedance changes can influence extracellular ionic currents, which are critical for generating local field potentials (LFPs) and facilitating ephaptic coupling between neurons. By providing a means to infer ECS dynamics, impedance measurements offer valuable insights into the mechanisms underlying neural communication and LFP modulation.

The integration of bio-impedance data into brain stimulation protocols further underscores its clinical and research utility. Impedance measurements help optimize electrical brain stimulation (EBS) by providing information on how impedance varies across different brain regions and states. This knowledge allows for the fine-tuning of stimulation parameters, ensuring more effective targeting of specific neural circuits while minimizing undesired side effects. Understanding impedance dynamics enables researchers to develop tailored stimulation strategies that account for regional and state-dependent variations in tissue conductivity and permittivity, ultimately improving the efficacy of EBS interventions.

In summary, bio-impedance, when combined with other bio-signals, provides a powerful and comprehensive approach for monitoring physiological processes such as epilepsy, sleep-related cerebral hemodynamics, and ECS dynamics. Its versatility extends to optimizing brain stimulation techniques, underscoring its potential for advancing both clinical applications and fundamental neuroscience research.

Table 5 builds on the references we mentioned in this section.

EIT (Electrical Impedance Tomography) is a portable, low-cost, and safe brain imaging technology that measures electrical impedance in tissues. However, its precision is relatively low. fcPAT (functional connectivity photoacoustic tomography) is another portable, low-cost, and safe brain imaging technique with high precision. EEG (electroencephalography) measures electrical signals in the brain. It is a medium-cost, wearable, and maneuverable technique with no side effects. CT (computed tomography) uses X-ray absorption to image tissues and is a high-cost, stationary technique with minimal side effects. MRI (magnetic resonance imaging) utilizes nuclear magnetic resonance and is a high-cost, stationary technique with minimal side effects. PET (positron emission tomography) measures radioactive tracer distribution in tissues. It is a high-cost, stationary technique with moderate side effects. The combination of EEG and EIT may offer a powerful tool for studying brain function and dysfunction. For instance, simultaneous EEG and EIT recordings can be used to investigate the relationship between electrical activity and impedance changes in the brain during seizures. By analyzing the spatial and temporal patterns of these signals, researchers can gain insights into the underlying mechanisms of epilepsy and potentially develop more effective diagnostic and treatment strategies. In addition, the use of EEG data in conjunction with EIT can improve the accuracy of image reconstruction and reduce the impact of artifacts. This integrated approach holds promise for advancing the field of brain imaging and improving patient care.

### 7.5. Neuromorphic Computing and Bio-Impedance: A Synergistic Approach for Intelligent Wearable Devices

Neuromorphic computing is transforming the way intelligent devices process information. Inspired by the human brain, it moves beyond traditional computing’s step-by-step approach and instead uses spiking neural networks to handle data in a more natural, event-driven way. This allows for real-time, parallel processing while using significantly less energy.

By integrating this technology into wearable devices, we can make them smarter and more efficient, enabling faster decision-making, longer battery life, and the ability to adapt and learn from their environment. In short, neuromorphic computing paves the way for a new generation of intelligent, low-power devices that think and respond more like the human brain, making technology more seamless and responsive than ever before. However, one of the biggest challenges in neuromorphic computing is the limited availability of input data for training and adaptive learning. Without sufficient and high-quality data, the system struggles to improve its accuracy over time.

One promising solution is integrating bio-impedance data—an important physiological measurement of tissue properties—alongside other electrical bio-signals such as EEG, ECG, and EMG. Combining these signals can provide a more comprehensive understanding of the body’s functions, significantly improving detection and prediction capabilities, particularly for brain disorders. This approach has the potential to enhance neuromorphic systems’ ability to recognize patterns, make decisions, and adapt more effectively, paving the way for smarter, more responsive healthcare technologies.

## Figures and Tables

**Table 2:** Ion concentrations in mequiv./L of ECF (plasma + interstitial) and ICF of muscle cells [69].

Electrolyte	Na^+^	K^+^	Ca^2+^	Mg^+^	Cl^−^	${\mathrm{HCO}}_{3}^{-}$	${\mathrm{PO}}_{4}^{-}$	Protein	Org Acid
Plasma	142	4	5	3	103	27	2	16	5
Interstitial	151	4.3	5.4	3.2	109.7	28.7	2.1	17	5.3
ICW muscle	10	160		35	2	8	140	55	

Adapted from Pitts [69].

**Table 3:** Comparison of different phenomena in brain impedance.

Phenomenon	Epileptic Seizures	Acute Stroke	Tumor	Hemorrhagic	Ischemic
Time Variation	Slow	Slow	Slow	Slow	Slow
Impedance Variation	Increase [29]	Decrease/Increase [95]	Decrease [84]	Decrease [71,93]	Increase [71,93]
ΔZ	10–12% [88]	-	6–7.5 × conductivity [84]	10–60% [30,94]	20–200% [30,89]
Brain Region	Focal ^1^	Focal/Global ^2^	Focal	Focal/Global ^2^	Focal/Global

^1^ Focal means regions strongly involved in the phenomenon exhibited significant impedance shifts. ^2^ Global means this phenomenon can potentially exhibit significant impedance shifts in all brain regions.

**Table 4:** Features and comparison of wearable electrodes, highlighting the differences between dry and wet types.

Category	Sub-Type	Pros	Cons
Wearable Electrodes	General Features	Non-invasive Portable and convenient Real-time monitoring Long-term suitability	Motion-induced accuracy reduction Less spatial resolution
	Wet Electrodes	More stable signals Higher-quality signal acquisition	Dry out over time
	Dry Electrodes	User-friendly and reusable Suitable for wearables Continuous monitoring	Higher impedance Sensitive to motion artifacts

**Table 5:** Brain imaging technology comparison.

Technique	Mechanism of Operation	Cost	Wearable	Operability	Side Effects	Precision	Relationship with Bio-Impedance	Diagnosable Diseases
EIT	Electrical impedance	L	Yes	Maneuverable	No	L	EIT and EEG can be used simultaneously to monitor brain activity and improve the localization of brain events, such as seizures.	Epilepsy, stroke, brain tumors, brain edema
fcPAT	Optical excitation and acoustic detection	L	Yes	Maneuverable	No	H	fcPAT is a complementary technique to EEG, providing information about hemodynamic changes associated with brain activity.	Functional brain imaging, brain disorders
EEG	Electrical signals in the brain	M	Yes	Maneuverable	No	M	EEG measures electrical activity in the brain, while bio-impedance measures the opposition to the flow of an alternating electrical current through brain tissues.	Epilepsy, sleep disorders, brain death, coma, encephalopathies, brain tumors, stroke
CT	X-ray absorption by tissues	H	No	Stationary	Minimal	H	CT provides structural information about the brain, which can be used to interpret bio-impedance changes.	Brain tumors, stroke, head injuries
MRI	Nuclear magnetic resonance	H	No	Stationary	Minimal	H	MRI provides high-resolution structural information about the brain, which can be used to interpret bio-impedance changes.	Brain tumors, stroke, multiple sclerosis
PET	Radioactive tracer distribution	H	No	Stationary	Moderate	H	PET provides functional information about the brain, which can be used to interpret bio-impedance changes.	Brain tumors, Alzheimer’s disease, Parkinson’s disease

## Data Availability

No new data were created or analyzed in this study. Data sharing is not applicable to this article.

## Abbreviations

The following abbreviations are used in this manuscript:

EIT	Electrical Impedance Tomography
EIS	Electrical Impedance Spectroscopy
MFEIT	Multi-frequency EIT
ECS	Extracellular space
REG	rheoencephalography
CBF	Cerebral blood flow
CSF	Cerebrospinal fluid
TBI	Traumatic brain injury
SBI	Secondary brain injury
TCB	Transcranial bio-impedance
ICP	Intracranial pressure
BIM	Bio-impedance monitoring
ECG	Electrocardiogram
EEG	Electroencephalogram
ECF	Extracellular fluid
ICF	Intracellular fluid
BIS	Bio-Impedance Spectroscopy
BIA	Bio-Impedance Analysis
SF-BIA	Single frequency BIA
ICW	Intracellular water
ECW	Extracellular water
TBW	Total body water
BMI	Body mass index
CPE	Constant phase elements
ETI	Electrode tissue impedance
MRI	Magnetic resonance imaging
fMRI	Functional MRI
ICV	Intracranial volume
IQ	In-phase and quadrature
IA	Instrumentation amplifier
ADC	Analog-to-digital converter
OTA	Operational Transconductance Amplifier
SNR	Signal-to-noise ratio
FFT	Fast fourier transform
MRPDD	Magnitude-ratio and phase-difference detection
GPD	Gain-phase detector
SD	Synchronous detection
DLF	Digital loop filter
CG	Current generator
CM	Common-mode
TC	Transconductance
AE	Active electrodes
ANN	Artificial Neural Network
ML	Machine learning
DL	Deep learning
GAN	Generative Adversarial Network
SNN	Spiking neural network
SEEG	Stereoelectroencephalography
PSG	Polysomnography
EBS	Electrical brain stimulation
REM	Rapid eye movement
NREM	Non-REM
CT	Computed tomography
